# Comparative Analysis of the Secretome and Interactome of *Trypanosoma cruzi* and *Trypanosoma rangeli* Reveals Species Specific Immune Response Modulating Proteins

**DOI:** 10.3389/fimmu.2020.01774

**Published:** 2020-08-27

**Authors:** Renata Watanabe Costa, Marina Ferreira Batista, Isabela Meneghelli, Ramon Oliveira Vidal, Carlos Alcides Nájera, Ana Clara Mendes, Izabela Augusta Andrade-Lima, José Franco da Silveira, Luciano Rodrigo Lopes, Ludmila Rodrigues Pinto Ferreira, Fernando Antoneli, Diana Bahia

**Affiliations:** ^1^Departamento de Microbiologia, Imunologia e Parasitologia, Escola Paulista de Medicina, Universidade Federal de São Paulo, São Paulo, Brazil; ^2^Departamento de Genética, Ecologia e Evolução, Instituto de Ciências Biológicas, Universidade Federal de Minas Gerais, Belo Horizonte, Brazil; ^3^The Berlin Institute for Medical Systems Biology-Max Delbrück Center for Molecular Medicine in the Helmholtz Association in Berlin, Berlin, Germany; ^4^Laboratorio Nacional de Biociências (LNBio), Campinas, São Paulo, Brazil; ^5^Departamento de Informática em Saúde, Universidade Federal de São Paulo, São Paulo, Brazil; ^6^RNA Systems Biology Lab (RSBL), Departamento de Morfologia, Instituto de Ciências Biológicas, Universidade Federal de Minas Gerais, Belo Horizonte, Brazil

**Keywords:** interactome, secretome, IL17, IL15, *Trypanosoma rangeli*, *Trypanosoma cruzi*

## Abstract

Chagas disease, a zoonosis caused by the flagellate protozoan *Trypanosoma cruzi*, is a chronic and systemic parasitic infection that affects ~5–7 million people worldwide, mainly in Latin America. Chagas disease is an emerging public health problem due to the lack of vaccines and effective treatments. According to recent studies, several *T. cruzi* secreted proteins interact with the human host during cell invasion. Moreover, some comparative studies with *T. rangeli*, which is non-pathogenic in humans, have been performed to identify proteins directly involved in the pathogenesis of the disease. In this study, we present an integrated analysis of canonical putative secreted proteins (PSPs) from both species. Additionally, we propose an interactome with human host and gene family clusters, and a phylogenetic inference of a selected protein. In total, we identified 322 exclusively PSPs in *T. cruzi* and 202 in *T. rangeli*. Among the PSPs identified in *T. cruzi*, we found several trans-sialidases, mucins, MASPs, proteins with phospholipase 2 domains (PLA2-like), and proteins with Hsp70 domains (Hsp70-like) which have been previously characterized and demonstrated to be related to *T. cruzi* virulence. PSPs found in *T. rangeli* were related to protozoan metabolism, specifically carboxylases and phosphatases. Furthermore, we also identified PSPs that may interact with the human immune system, including heat shock and MASP proteins, but in a lower number compared to *T. cruzi*. Interestingly, we describe a hypothetical hybrid interactome of PSPs which reveals that *T. cruzi* secreted molecules may be down-regulating IL-17 whilst *T. rangeli* may enhance the production of IL-15. These results will pave the way for a better understanding of the pathophysiology of Chagas disease and may ultimately lead to the identification of molecular targets, such as key PSPs, that could be used to minimize the health outcomes of Chagas disease by modulating the immune response triggered by *T. cruzi* infection.

## Introduction

*Trypanosoma cruzi* and *Trypanosoma rangeli* belong to the genus *Trypanosoma* and the family *Trypanosomatidae*. They are the only two trypanosomes that infect humans in Latin America (1). *T. rangeli* and *T. cruzi* share the same mammalian hosts and triatomine vectors in overlapping areas. Although *T. rangeli* is non-pathogenic to mammalian hosts, it is harmful to insects. In particular, it causes morphological abnormalities or even lethal effects in molting and feeding in the genus *Rhodniu*s (2). It has been recently reported that *T. rangeli* is more closely related to Old World trypanosomes of bats, civets, rats, and monkeys than to *T. cruzi* (3). *T. cruzi* causes Chagas disease, which is endemic in Latin America. Chagas disease afflicts 6–7 million individuals worldwide and has been associated with negative economic impacts in developing countries (4). Approximately 28,000 new cases of Chagas disease are diagnosed and 12,000 deaths are reported every year (4), indicating that Chagas disease is still a relevant public health issue (5). In addition to these estimates, demographic and migratory changes have resulted in the spread of the disease to non-endemic areas in the American continents, including the United States, and to other continents (6). *T. rangeli* shares morphological similarity and immunological cross-reactivity with *T. cruzi*, and they can co-occur as natural mixed infections in both vertebrate hosts and insect vectors in a broad geographical area (7).

The genome of *T. rangeli* has been sequenced and compared to that of *T. cruzi* (8). It has been shown that *T. rangeli* contains a smaller number of gene copies of virulence factors encoded by multigene families such as the mucin-associated proteins (MASPs), trans-sialidases (TS), and mucins; and a reduced repertoire of genes encoding antioxidant enzymes compared to *T. cruzi* (8). Additionally, transcriptomic analysis showed that *T. rangeli* contains genes encoding factors of virulence and pathogenicity, such as gp63, sialidases, and oligopeptidases, as has been described in other kinetoplastids (9). Proteins that are secreted to the extracellular medium usually contain an N-terminal signal peptide (SP) that drives them to the classical endoplasmic reticulum (ER)/Golgi-dependent secretion pathway [reviewed in Watanabe Costa et al. (10)]. In addition, other proteins without the canonical SP are secreted via non-classical pathways, usually through the shedding of extracellular vesicles (11). A significant amount of data on the genome structure and expression of human parasitic trypanosomes is currently available. There have been many studies of the biological functions of *T. cruzi* secreted proteins and their role in parasite-host interactions and pathogenesis [reviewed in Watanabe Costa et al. (10)].

As it is possible that secreted molecules from trypanosomes modulate host pathways including the immune response, the present study focused on species-specific potentially secreted proteins (PSPs) from *T. cruzi* and *T. rangeli* and their interaction with the host immune response. Using a computational pipeline which integrates genomic and proteomic data with bioinformatics predictions, we built an *in silico* secretome of both species and identified classically PSPs (i.e., those displaying the SP without transmembrane domains). Additionally, we built an interactome and evolution models of a few selected PSPs. This comparative analysis of *T. cruzi* and *T. rangeli* may provide new insights into how these sympatric species evolved and adapted to the mammalian host.

## Materials and Methods

### Protein Sequences of *Trypanosoma cruzi* and *T. rangeli*

The protein sequences of *T. cruzi* (*T. cruzi* Sylvio X10/1-2012) used in this study were acquired from the TriTrypDB kinetoplast database. The protein sequences in FASTA format of *T. cruzi* was downloaded in January 14th 2015 and September 20th 2019. The protein sequence of *T. rangeli* was provided by the Laboratory of Bioinformatics of the LNCC—National Laboratory of Scientific Computing and deposited in TriTrypDB (12) (*T. rangeli* SC58). Both sequences contained automatic annotations of proteins.

### Prediction and Selection of PSPs

SignalP (version 4.1; default parameters) (13) was used to predict the presence and location of signal peptide (SP) cleavage in the amino acid sequence. SecretomeP (version 1.0; default parameters) (14) was used to perform predictions of secreted proteins by the non-classical pathway (lacking the canonical SP). In addition to SignalP, SecretomeP predicts arginine and lysine cleavage sites in eukaryotic protein sequences and subcellular localization.

In the case of the parasites studied here, there were some proteins with a SP, but also with additional transmembrane regions (which may belong to the cytoplasmic membrane or to the nuclear membrane, for example). Therefore, the program TMHMM (version 2.0) (15) was used to perform prediction of transmembrane helices. HMMTOP (version 2.0) (16, 17), Tmpred (18), and DAS-Tmfilter (19) were used to validate the prediction from TMHMM.

This prediction was done by the following steps: (i) generate fasta files of clean protein sequence (with start metionine and translated stop codon) with proteins for the individual species, (ii) run the SignalP and SecretomeP pipelines, (iii) run the TMHMM program to identify transmembrane proteins, (iv) validate the TMHMM data with online programs (HMMTOP 2.0, Tmpred, and DAS-TMfilter). The final result included only proteins with SP lacking other additional transmembrane regions. The steps of the pipeline performed are shown in Supplementary Figure 1.

### Annotation of PSPs

The second part of the analysis consisted of evaluating the results from the protein prediction programs and confirming them through computational and manual inspection. In addition, the Blast2Go (20) (https://www.blast2go.com/) also provided additional information for proteins noted as hypothetical, helping the manual inspection.

The BlastGO annotations were based on the similarity levels of the local file sequences with the QBlast database (21). Mapping parameters were changed to search results on the non-redundant reference protein (nr) (21), PSD (21), UniProt (22), Swiss-Prot (23), TrEMBL (23), RefSeq (24), GenPept (21), and PDB (25). Assignment of functional terms of the set of GO terms was assembled in the mapping step. Pie charts were used for showing the biological functions of *T. cruzi* and *T. rangeli*.

In the case of proteins annotated as hypothetical in TriTrypDB for both species, we used the annotation of the InterPro (26) and domain entries were characterized if available. InterPro is embedded in TriTrypDB (https://tritrypdb.org/tritrypdb/showQuestion.do?questionFullName=GeneQuestions.GenesByInterproDomain) and once you insert the Gene ID to search an automatic page opens and the features of the sequence are shown, including “13. Protein features and properties” where there is the option of Interpro domains.

### Gene Family Comparative Analysis

OrthoMCL (version 1.4) (27–29) (https://orthomcl.org/orthomcl/?rm=orthomcl) was used to verify which PSPs were expanded in both species. FASTA sequences of PSPs of *T. cruzi* and *T. rangeli* were used in the comparative analysis. In summary, OrthoMCL compares all proteins against all protein sequence similarities and find clusters based on reciprocal similarities. Protein clusters exhibiting bidirectional similarities between at least two *Trypanosoma* species were considered orthologs and those with bidirectional similarities within each species were classified as paralogues.

### Criteria for Selection of PSPs for Interactome Analysis

From the OrthoMCL results, some proteins were selected for both phylogeny and interactome analyses. The following selection criteria were used: (a) potential modulators of the host immune system were selected based on the available protein annotation in the results of Blast2Go similarity annotation and literature survey of each protein. For the hypothetical or unknown proteins, conserved domains in other species were investigated to infer their characterization in NCBI databases, such as Pfam (*European Bioinformatics Institute*) (30); (b) some degree of identity with proteins of the human host. The sequences were individually verified in the BLAST (nr database) with the “Organism” field filled with *Homo sapiens*. The degree of identity can be visualized in the search result by descending the “Ident” column. Proteins sharing homology with the human proteins were considered and those that did not share homology were withdrawn from the interactome analyzes; (c) annotations not yet determined in databases. Hypothetical or unknown proteins were prioritized in the analysis because of the importance of exploiting the proteins not yet well-documented in the literature. To obtain some basic data of these proteins as biological functions, the conserved domains with proteins of other species were investigated; and (d) proteins found in both species and those present in only one species. For this criterion, OrthoMCL data were used to generate a Venn diagram containing proteins of both species at the intersection with proteins present only in each species outside the intersection.

These criteria allowed us to elaborate a comparative analysis between PSPs of *T. rangeli* and *T. cruzi* and to identify virulence-related proteins of *T. cruzi*, possibly interacting with the human immune system.

### Phylogenetic Inference

*T. cruzi* phospholipase A2 (PLA2) was selected to perform phylogeny inference. Sequences were obtained by a search using MEGABLAST (31) (search for highly similar sequences) from patatin protein domain from PLA2 (phospholipase 2) of *Trypanosoma cruzi* Sylvio strain as query, resulting in a set of 4,354 sequences (Supplementary Table 6).

We obtained multiple sequence alignment by Muscle algorithm (32), included in the Seaview software (33), based on 4,354 patatin domain protein sequences of PLA2 (phospholipase 2). Phylogenetic relationships analyses were made by neighbor-joining (NJ) and Bayesian methods. For NJ trees the confidence scores of each node were assessed with bootstrap analysis (34), with 100 replicates. Bayesian inference was performed using MrBayes 3.2 software (35) based on a reduced set of PLA2 patatin sequences, a set with 78 sequences aligned by Muscle (36) with Γ distribution (LG+G) to correct for rate variation among sites was selected as the substitution model to Bayesian inference (Supplementary Table 7). Substitution models were chosen based on the Akaike Information Criterion (AIC) and Bayesian Information Criterion (BIC), using MEGA 6 software (“find best DNA model” option) (37). Bayesian consensus tree was constructed with four million generations with sampling every 100 generations until the standard deviation from split frequencies were under 0.01. Phylogenetic trees figure edition was then formatted with the FigTree v1.3.1 software (http://tree.bio.ed.ac.uk/software/figtree/).

### Gene and Pathway Network Analysis

The software Ingenuity Pathways Analysis (IPA) (Qiagen, USA—www.ingenuity.com) was used to build gene and pathways interaction networks for the parasite PSPs identified by a screening for orthologous in human protein databases. Proteins were selected for human interactome according the criteria cited above (2.5): EKG03115.1 chaperone DNAJ protein, putative [*Trypanosoma cruzi*], TCSYLVIO_005847; and two heat-shock HslVU, ATPase subunit HslU [*Trypanosoma rangeli* SC58], TRSC58_01986 and TRSC58_04483 and a 4-nitrophenyl phosphatase, TRSC58_05352. The list of proteins used in the analysis were as follows: *T. rangelli*: XP_011519466.1, BAF85005.1, BAF85005.1, NP_006651.2, NP_006651.2, CAB66856.1, CAB99462.1 and *T. cruzi*: BAC05229.1, NP_061072.3, AAO31694.1, NP_005871.1, NP_001530.1, BAA02656.1, AAB69313.1, EAW58527.1, NP_001123655.1, NP_057390.1, CAG33377.1, AAK69110.1, EAW85303.1, AAH31044.2. IPA maintains a graphical database of networks of interacting genes (Ingenuity Knowledge Base, IKB, Qiagen, USA-www.ingenuity.com) the analysis performed was based on the content of date 2019-05. Molecules are represented as nodes, and the biological relationship between two nodes is represented as an edge (line). All edges are supported by at least one reference from the literature, from a textbook, or from canonical information stored in the IK.

## Results and Discussion

### Identification of the Potentially Secreted Proteins (PSPs) of *T. cruzi* and *T. rangeli*

**Figure 1:**
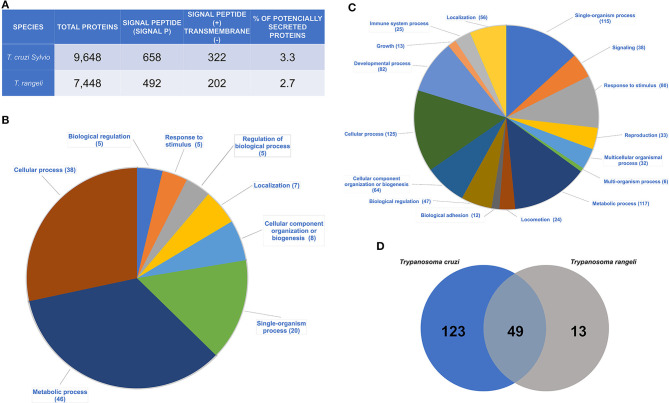
Identification of the potentially secreted proteins (PSPs) of *T. cruzi* and *T. rangeli*. **(A)** Trypanosome species analyzed. Number of total protein sequences, amount of protein sequences with signal peptide [SIGNAL PEPTIDE (+)] and without transmembrane domains [TRANSMEMBRANE (-)]. Percentage of PSPs identified from the genome of each species; **(B)** Biological processes of the *T. cruzi* PSPs found by the Blast2Go program. The numbers given in parentheses show the amount of proteins related to a given biological function; **(C)** Biological processes of the *T. rangeli* PSPs found by the Blast2Go program. The numbers given in parentheses show the amount of proteins related to a given biological function; **(D)** Quantitative analysis by OrthoMCL. Amount of grouped proteins found in each organism and in both species.

Of the 10,794 predicted protein sequences from the *T. cruzi* Sylvio X10/1 genome, 658 sequences were found to contain the canonical SP and 322 (~49%) among them did not contain transmembrane helices according the algorithm TMHMM2.0 (Figure 1A, Supplementary Table 1). These latter sequences were here categorized as potentially secreted or secretory proteins (PSPs) that are targeted to the endoplasmic reticulum by the signal recognition particle (SRP)-receptor pathway. Among 7,448 proteins of the *T. rangeli* SC58, 492 sequences had the SP and 202 (41%) of these sequences presented the SP without transmembrane helices (Figure 1A, Supplementary Table 1). Comparing the number of PSPs from *T. cruzi* and *T. rangeli*, we observed that the percentage of PSPs was very similar in both species. Only 3.3 and 2.7% of total protein sequences of *T. cruzi* and *T. rangeli* corresponded to the PSPs, respectively (Figure 1A). Among the SP*-*bearing proteins, those containing transmembrane loops were discarded because they could be targeted to the membranes of other cell organelles such as nuclear and mitochondrial membranes or they are cell surface membrane -spanning proteins. It is noteworthy that among PSPs (Figure 1A) there are membrane associated proteins by other post-translational modifications or membrane bound proteins (38).

### Annotations and Biological Functions of PSPs

Among the PSPs, most proteins of *T. cruzi* were annotated as hypothetical followed by a group of *T. cruzi* multigene families such as mucin-associated surface proteins (MASP), mucin (TcMUCII), trans-sialidases and trans-sialidases-like (TS), RHS, gp63 surface protease, and DGF-1 (Supplementary Table 2, Supplementary Figure 2). Single copy genes code most of hypothetical proteins classified as PSPs in *T. cruzi*, whereas few PSPs (e.g., TCSYLVIO_011009; TCSYLVIO_008005) belong to small gene families (15–30 members). Other proteins were found in a smaller number in the analysis (Supplementary Table 2, Supplementary Figure 2). Finally, the main biological functions related to the *T. cruzi* PSPs were cellular, metabolic and single-organism processes (Figure 1B, and to detailed functional categories of PSPs see Supplementary Table 2).

Concerning *T. rangeli* PSPs, cellular and metabolic processes are the main biological functions (Figure 1C, Supplementary Figure 2, and to detailed functional categories of PSPs see Supplementary Table 3). Other proteins were involved in regulation of biological processes, such as response to stimuli, location, and organization of cellular components and biogenesis (Figure 1C, and to detailed functional categories of PSPs see Supplementary Table 3).

### Comparative Analysis of OrthoMCL Cluster Composition

Using OrthoMCL software, protein clusters (families) were identified for *T. cruzi* and *T. rangeli*. A total of 123 PSPs were present only in *T. cruzi*, 49 proteins were shared by both species, and 13 proteins were found only in *T. rangeli* (Figure 1D, Table 1, Supplementary Table 4).

**Table 1:** Qualitative data of protein families found by OrthoMCL (full table in Supplementary Table 4).

***Trypanosoma cruzi* Sylvio**	**Both species**	***Trypanosoma rangeli***
Trans-sialidase	Surface protease GP63/Leishmanolysin	**Heat shock protein HslVU, ATPase subunit HslU**
Mucin TcMUCII	Glucose-regulated protein 78	4-nitrophenyl phosphatase
Retrotransposon hot spot	Serine/threonine protein phosphatase	^*^HP: mucin-associated surface protein (MASP)
Mucin-associated surface protein (MASP)	Methyltransferase	^*^HP: phosphoenolpyruvate carboxykinase
Dispersed gene family protein 1 (DGF-1)	DNA ligase	^*^HP: WD40 domain
90 kDa surface protein	Cytochrome-b5 reductase	Hypothetical protein
Surface protease GP63	Legume-like lectin	
Lipase	NADH-cytochrome b5 reductase	
Protein disulfide isomerase	RNA editing complex protein MP46	
Aldehyde dehydrogenase	ATP-dependent DEAD/H RNA helicase	
UDP-Gal or UDP-GlcNAc- dependent glycosyltransferase	Dihydrolipoamide acetyltransferase	
Surface protein ToIT	Acid phosphatase	
**Heat shock protein DNAJ**	Cyclophilin	
Hypothetical protein	^*^HP: DNA-binding transcriptional activator domain	
	^*^HP: NAD(P)-binding domain	
	FAD-dependente oxidoreductase domain	
	^*^HP: heat shock 70 kDa protein domain	
	^*^HP: LD-Carboxypeptidase domain	
	Hypothetical protein	

* *HP, hypothetical protein*.

In comparison to *T. rangeli*, we observed a higher number of genes belonging to multigene families in *T. cruzi*, such as mucins (TcMUCII), MASPs, and TS/TS-like proteins (39–47) (Table 1, Supplementary Table 4); and dispersed gene family protein 1 (DGF-1) (40, 48–52), gp63 (40, 53–56), and gp90, a putative 90 kDa surface protein which was renamed SAP (Serine, Alanine, and Proline-rich protein) (57, 58). Stoco et al. (8) demonstrated that the *T. rangeli* genome contains a smaller number of sequence copies from MASP, mucin, and TS/TS like proteins compared to the *T. cruzi* genome (8), which is consistent with our PSP findings. All these proteins have been reported to participate in the host invasion process, including virulence and pathogenicity. The MASP family is the second largest *T. cruzi* family of proteins, representing ~6% of the parasite genome (40, 41). MASPs may be involved in lymphocyte activation, promoting polyclonal expansion, and hypergammaglobulinemia, which delays the specific humoral response characteristic of the acute phase of Chagas disease. B cell activation promotes the immune response, preventing the specific response from occurring, preventing parasite neutralization and elimination (59–62). Studies have shown that TS/TS-like proteins are virulence factors, as they are involved in the adhesion of *T. cruzi* to the host cell, internalization, and intracellular survival [reviewed in (63)]. *T. cruzi* mucins (TcMUCII) found in the present study are present on the parasite surface and have two main functions: (i) provide protection against vector and host defense mechanisms and (ii) ensure the target for invasion into specific cells or tissues (43, 64–67). Some studies have reported that the gp63 glycoproteins identified by this study have the ability to inactivate the host complement system, facilitating the invasion and survival of *T. cruzi* (54–56, 68). SAP binds to the host cell and induces intracellular Ca^2+^ mobilization and host cell lysosome exocytosis (57, 58).

Among the hypothetical proteins, we identified a domain from heat shock protein 70 (Hsp70) which is presented in both species (Supplementary Table 1, green lines). Hsp70 interacts with cells of the immune system, exerting immunoregulatory effects. Exogenously added Hsp70 has potent cytokine activity and binds with high affinity to the plasma membrane, triggering rapid intracellular Ca^2+^ flow, activating nuclear factor kβ (NF-kβ), and positively regulating proinflammatory cytokine expression in human monocytes (69). From a diagnostic point of view, although anti-Hsp antibodies are unable to distinguish chagasic patients from those infected with other trypanosomes, *T. cruzi* anti-Hsp70 antibodies can distinguish between healthy and infected patients and between those in the acute phase and those in the chronic phase (70, 71). These results, in combination with the findings of our study, corroborate the importance of studying *T. cruzi* secreted proteins.

Several PSPs were found in both species, including gp63 surface proteases, glucose-regulated proteins, serine/threonine phosphatases, methyltransferases, DNA ligases, cytochrome b5 reductase, acetyltransferases, and others (Table 1, Supplementary Tables 1, 4). These proteins are related to cellular processes, replication, and metabolism. Among these proteins, only gp63 has been described in previous studies as being related to *T. cruzi* infection in host cells. Gp63 is differentially expressed in specific stages of the parasite cycle and is more highly expressed in amastigotes than in epimastigotes or trypomastigotes (72). Previous genomic studies have demonstrated that despite the non-pathogenic nature of *T. rangeli* in mammals, several secreted, and surface protein genes associated with virulence and pathogenicity in other pathogenic trypanosomatids, such as gp63, are present in *T. rangeli* (8, 73) (Table 1, Supplementary Tables 1, 4). Finally, in the group of protein families found only in *T. rangeli*, some hypothetical proteins were grouped with domains in MASPs, WD40, and phosphoenolpyruvate carboxylase, in addition to phosphatases and heat shock proteins HsIVU (Table 1, Supplementary Table 4).

Interestingly, some SP-displaying proteins already described to be secreted in the CL Brener clone, such as cruzipain (TcCLB.507603.270, major cysteine proteinase, putative, Esmeraldo-like; TcCLB.507603.260, cysteine peptidase, putative, Esmeraldo-like), P21 (TcCLB.509767.140, Esmeraldo-like) [both reviewed in Watanabe Costa et al. (10)], and mevalonate kinase (TcCLB.436521.9, Esmeraldo-like) (74, 75) were not found among the PSPs in the Sylvio clone (X10/1-2012) secretome (Supplementary Table 1). A similarity search (BLAST software) using tags of CL Brener in TriTrypDB did not find similar molecules exhibiting SPs in *T. cruzi* Sylvio (X10/1-2012). However, phospholipase and cyclophilin (10) do appear in *T. cruzi* Sylvio (X10/1-2012) secretome searches. These differences may be due to the high polymorphism among *T cruzi* strains from different lineages (CL Brener- lineage TcVI and Sylvio X10/1-lineage TcI), which is likely reflected in their infectivity modeling in different hosts, pathology evolution, and clinical manifestations (76). Conversely, a biological secretome (77) of *T. cruzi* CL Brener showed that many proteins found using our *in silico* strategy are also found in the biological secretome, such as protein kinases, lipases, heat shock proteins, DNA repair proteins, superoxide dismutase, transialidases, gp63, mucins, MASPs, and DGF-1. This indicates that *in silico* strategies are useful in selecting PSPs before proceeding to subsequent biological studies.

To find orthologs for PSPs in other isolates of *T. cruzi* we performed a manual search with BLASTP using a sample of 186 PSPs of Sylvio X10/1 as query against the genomes of clones Dm28 (TcI), CL Brener (TcVI, Esmeraldo-like, and non-Esmeraldo-like haplotypes) and TCC (TcVI) deposited in TriTrypDB (Supplementary Table 5). One hundred and three orthologous (55.4%) found in these isolates can be classified as PSP according our criteria. They are distributed as follows, 45 PSPs (24.2%) were found in the three isolates, whereas 58 PSPs (31.2%) were found in one or two isolates. Eighty-three proteins (44.6%) could not be classified in any of the three isolates. The variability regarding the presence of SP can be noticed when comparing the PSPs of *T. cruzi* Sylvio X10/1 (TcI) by BLAST with orthologs of other strains from the same TcI lineage, such as Dm28, or clones CL Brener e TCC from a different lineage (TcVI).

### Characteristics of Proteins Selected for Phylogeny and Interactome Analyzes

After OrthoMCL analysis, some proteins have been selected to perform both phylogeny inference and interactome analyzes (see Materials and Methods: 2.5. Criteria for selection of PSPs for interactome analysis), taking into account that the proteins selected must indicate implications in the modulation of host immune system.

TCSYLVIO_003749 (Supplementary Table 1, yellow line), annotated as a hypothetical protein, was selected only for phylogeny, because it did not have any similarity to human proteins to perform interactome analysis. It is a hypothetical PSP with a phospholipase 2 (PLA2) domain found in *T. cruzi* Sylvio. This protein was also found in *T*. *cruzi* CL Brener, exhibiting a SP but no transmembrane regions (TcCLB.506705.40). Although this protein did not meet protein filter criteria “b” (see Materials and Methods: Criteria for selection of PSPs for interactome analysis) previous studies have shown a relationship between *T. cruzi* phospholipases and parasite invasion and survival in the host (78, 79). In *T. rangeli*, the orthologous protein (TRSC58_01394) has transmembrane regions, thus it was not included in the secretome list. It is a hypothetical triacylglycerol lipase 3 (TGL3) carrying the conserved protein domains of superfamilies DUF 3336, patatin, and phospholipase A2 (PLA2). This is referred to as PLA2-like in later analyses. TGL3 is reported to be responsible for triacylglycerol lipase activity in the lipid particle. Patatin is a family consisting of several plant glycoproteins (80). The phospholipases present in *T. cruzi* could be related to host immune evasion mechanisms. It has been shown in Vero cells that phospholipase A1 is involved in cellular lipid modifications leading to protein kinase C (PKC) activation. With PKC activation, Ca^2+^ release occurs from intracellular stocks, contributing to parasite invasion (79).

DNAJ (TCSYLVIO_005847) (Table 1, Supplementary Table 1, blue lines, Supplementary Table 4), was found to be a PSP only in *T. cruzi* and was selected to build a hypothetical hybrid interactome, as it has homology with human proteins. *T. cruzi* DNAJ (or Hsp40) protein, acts as a Hsp70 chaperone forming a protein folding pathway that integrates with Hsp90. Several environmental stimuli acting upon the parasite during evolutionary selection have resulted in a very expanded and varied chaperone network. Heat shock protein expression is increased during the transition of the parasite from the vector insect (temperature ~ 26°C) to the human host (~ 37–38°C), during which there is differentiation from trypomastigotes to amastigotes. Heat shock proteins are also involved with pathogenesis in the host. For example, Hsp90, paralleling the human interactome proteins HSP90AB1 and HSP90AA1, may be involved in the development and intracellular growth of the parasite and represents a potential target for therapeutic interventions (81–84). Real et al. (85) demonstrated that *Leishmania (L.) amazonensis* (trypanosomatid that causes Leishmaniasis) secretes an ortholog to mouse Hsp70 kDa protein 5 (HSPA5) (69% identity; 90% sequence coverage). Considering that parasite factors mimic their mammalian counterparts, a hypothetical *Leishmania*-mouse interactome was proposed to identify host components that could be affected by the secretion of Leishmania HSPA5. The model predicted an interaction between an *L. amazonensis* HSPA5 and mammalian Toll-like receptor 9, which is implicated in important immune responses such as cytokine and nitric oxide production (85).

We selected three proteins found only in *T. rangeli* by OrthoMCL (Table 1, Supplementary Table 1, blue lines, Supplementary Table 4) that share homology with human proteins: two heat shock HsIVU (TRSC58_01986 and TRSC58_04483) and a 4-nitrophenyl phosphatase, TRSC58_05352. They are present in several strains of *T. cruzi* (e.g., CL Brener), but they do not have SPs as they do in *T. rangeli*. According to BLAST, proteins TRSC58_01986 and TRSC58_04483 (heat shock domain HsIVU) have homology with some human proteins, such as ATPases and ATP-dependent metalloproteinases, and TRSC58_05352 (4-nitrophenyl phosphatase) has homology to some human phosphatases.

### PLA2, a Possible Immune Modulator, Has a Plant Phospholipase Domain Which Has Not Been Acquired by Horizontal Transfer

Phospholipases are involved in multiple physiological processes, including the generation of lipid signaling. Tc-PLA1, *T. cruzi* phospholipase A1, is secreted into the extracellular medium during the infective stages (amastigotes and trypomastigotes). It displays high membrane binding activity and may be involved in parasite-host interaction events prior to cellular invasion (78, 79). Interestingly, Tc-PLA1 does not display a SP in the Sylvio strain, but does in the CL Brener strain. We then chose the hypothetical protein PLA2, another phospholipase, to generate a phylogenetic tree because of a potential function in modulating host immune mechanisms and parasite invasion due to a similar function in PLA1 and the existence of a Patatin-like domain in its sequence.

Patatin is a family of glycoproteins that accounts for up to 40% of the total soluble protein in potato tubers (86). When we performed a BLAST search with PLA2-like of *T. cruzi* (TCSYLVIO_003749), many plant species sequences with some similarity to the Patatin domain were retrieved (not shown). Because of this, we questioned whether the Patatin domain in the PLA2-like gene had been acquired by horizontal transfer (HTG). We have reported an HTG event of a FYVE domain present only in viruses, such as *Acanthamoeba mimivirus*, transferred to phosphatidylinositol kinases (PIK) of *T. brucei, Leishmania*, and *T. cruzi* (87). The FYVE-PIK architecture is only present in trypanosomatids and viruses, suggesting a horizontal acquisition hypothesis that was supported by Bayesian phylogenetic inference (87). An HTG event has also been demonstrated for some *Trypanosoma* species in which they acquired Proline-racemase (PRAC) genes, previously identified in a restricted group of bacteria. The PRAC genes act as virulence factors in the highly pathogenic bacteria *Clostridium difficile* and *Pseudomonas aeruginosa* (88).

**Figure 2:**
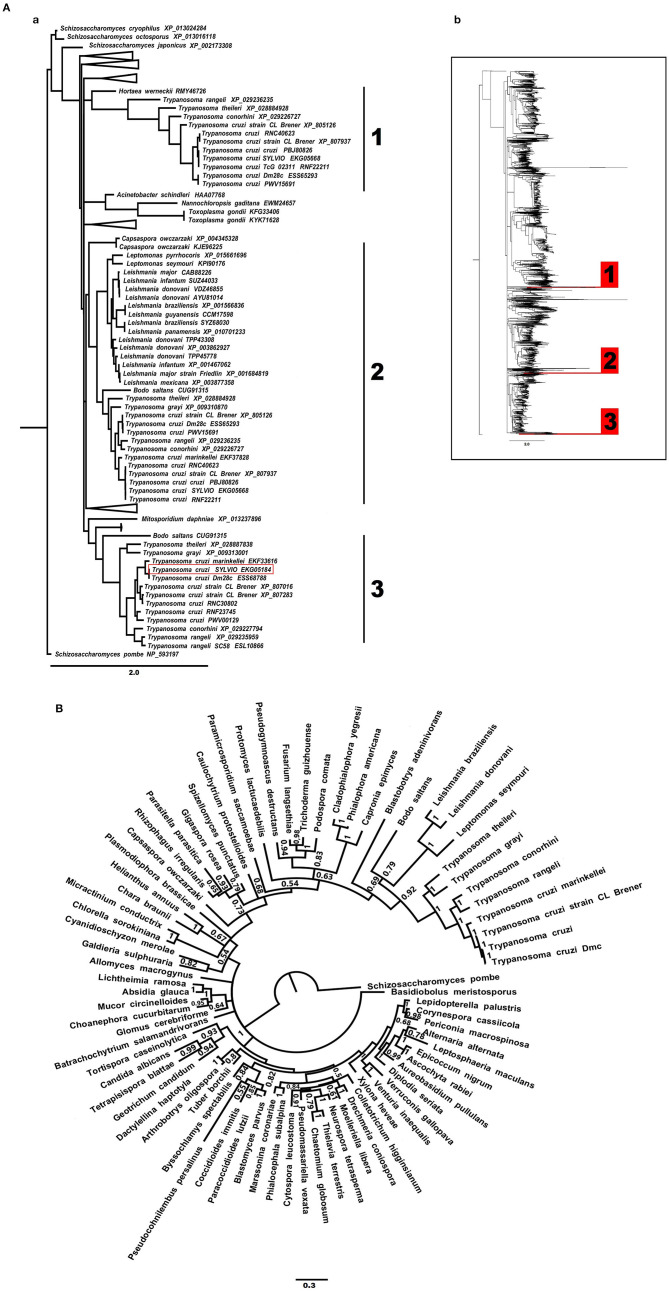
Patatin phospholipase domain has not been acquired by horizontal transfer (HGT). **(A)** Neighbor-joining (NJ) phylogenetic analysis of PLA2 patatin protein motif sequences from several species. The phylogenetic tree was constructed using 4,354 amino acid sequences aligned by Muscle with NJ algorithm, provided with Seaview. The branch length is proportional to amino acid differences. **(a)** NJ phylogenetic edited tree showing three distinct *Trypanosoma* species branches. SYLVIO strain EKG05184 (used as the query in the BLAST search) is highlighted, other species are included in collapsed branches. **(b)** Global NJ phylogenetic tree with all 4,354 species; three *Trypanosoma* branches are highlighted. There were no distant species inserted in *Trypanosoma* branches indicating HGT. **(B)** Bayesian tree constructed based on a set with 78 PLA2 patatin protein motif sequences. Node labels represent the posterior probabilities values. There were no distant species in *Trypanosoma* branches indicating HGT.

Thus, to test the hypothesis of PLA2-like patatin domain HGT, we generated a phylogenetic gene tree based on PLA2-like amino acid sequences. While, “gene trees” represent the evolutionary history of the genes studied and can be incongruent with species trees, “species trees” are usually constructed with orthologous genes, aiming to recover the genealogy of taxa (89). The reason for the incongruence can be: (1) differences in rates of evolution, (2) occurrence of gene loss and/or gene duplication, (3) recombination between neighboring regions, and (4) horizontal gene transfer (HGT) (90). The use of phylogeny can indicate HGT events by revealing incompatibility between the gene and species evolutionary histories exposed by reconstruction of the phylogenetic gene and species trees, using the species tree as a reference (89). Differences between gene trees and species trees can be suggestive of HGT events. Gene trees can provide evidence for genetic processes as well as HGT. If two species are connected in the same branch of a gene tree but are evolutionarily distant according to the species tree, a HGT event has possibly occurred (91). Inference of horizontal gene or sequence transfer by phylogenetics is considered more sensitive and specific compared to other approaches (92). We constructed a gene tree based on the patatin protein domain from PLA2 (phospholipase 2) to search for a possible HGT event that could have affected *T. cruzi*. Using the PLA2 patatin domain protein sequences from the *T. cruzi* Sylvio strain, we searched for similar sequences using MEGABLAST (31) (search for highly similar sequences). We obtained 4,354 sequences that were extracted from BLAST (21) (Supplementary Table 6) and aligned by Muscle into Seaview software (33). Subsequently, we generated a neighbor joining (NJ) phylogenetic tree (Figure 2A). The NJ tree generated three clusters with *Trypanosoma* species, but there was no one distant species/taxon insertion that could be an indicator of putative HGT. Three *Trypanosoma* clades, far from each other in the same tree, occurred due to variation in the BLAST results (*score* and *e-value*). We also conducted a Bayesian phylogenetic inference based on a restricted PLA2 patatin sequence alignment using MrBayes software v.3.2 (35), which confirmed the absence of HGT (Figure 2B).

### Hypothetical Hybrid Interactome Revealed Proteins Potentially Involved in Host Immune Modulation

**Figure 3:**
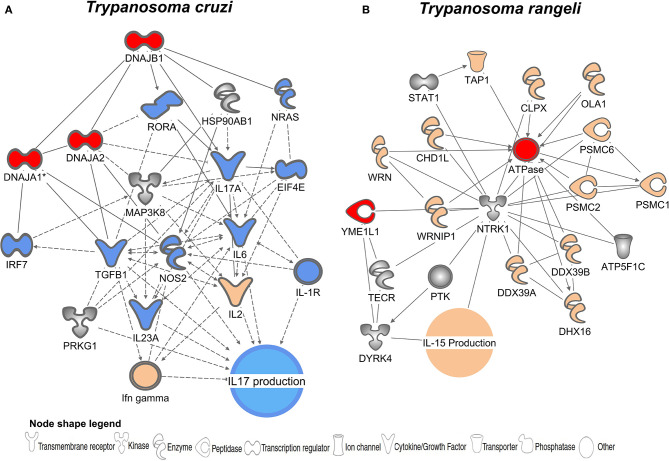
Hypothetical hybrid interactome revealed proteins potentially involved in host immune modulation. Gene and functional interaction networks for potential *T. cruzi* **(A)** and *T. rangeli* **(B)** PSPs (red nodes) identified by screening for orthologous genes in human protein databases. Molecules are represented as nodes (shaded gray), and the biological relationship between two nodes is represented as an edge (line). A line denotes binding of proteins, whereas a line with an arrow denotes that one protein acts on another. A dotted line denotes an indirect relationship. Each node shape represents one type of molecule, as described in the figure legend. Molecules in orange represent activation and those in blue represent inhibition. Colorless molecules indicate no prediction of interaction.

IPA (Ingenuity Pathway Analysis) was used to identify functional and molecular networks among the list of *T. cruzi* and *T. rangeli* PSPs and host molecules (Figures 3A,B). The analysis generated two different networks for the PSPs of *T. cruzi* and *T. rangeli*. The genes and gene products are represented as nodes, and the relationships between them are represented as edges (lines). All edges are supported by at least one literature citation or by canonical information stored in the database. Molecules were mapped to their corresponding node. The network generated from the *T. cruzi* PSPs (represented as red nodes) showed genes related to IL-17 production and showed central nodes including IL-17A and NOS2 (Figure 3A). For *T. rangeli*, IL-15 production was the main biological function predicted to be related to the genes in the network (Figure 3B).

*T. cruzi* induces expression of proteins that trigger a decrease in IL-17 production, while *T. rangeli* leads to interleukin 15 (IL-15) production, as shown in Figures 3A,B, respectively. Although both species are parasites of mammals and are evolutionarily related, *T. rangeli* infection in humans is promptly eliminated by the host (93–96). Studies have failed to demonstrate *T. rangeli* multiplication in vertebrate hosts, and its pathogenicity and virulence are still not well-understood (95–97). Thus, very little information is available regarding induction of the immune response by *T. rangeli* since this parasite appears to be harmless to mammals and may be eliminated by the complement system (98). Successful immune responses against pathogens depend upon efficient stimuli and consequent cytokine synthesis and equilibrium (99, 100). According to IPA analysis, *T. rangeli* triggers IL-15 synthesis, which is associated with nuclear factor (NF)-kB complex activation (Figure 3B). It has been demonstrated in cerebral epithelial cell lines that nuclear translocation of the p65 subunit of NF-kB is one of the major activators of IL-15 upon stimulation with TNF-α (101). IL-15 promotes B, T, and NK cell differentiation and proliferation and also functions as a danger signal during the inflammatory process (102, 103). Our data suggest that the inability of *T. rangeli* to establish infection may be related to the activation of IL-15. IL-15 signaling begins with the binding of IL-15 to IL15Rα on the surface of antigen-presenting cells, triggering *trans* presentation to effector cells through its heterotrimeric receptor (IL15Rα, IL-2R/IL-15Rβ, and the typical cytokine receptor y-chain) and can lead to activation of the immune response by diverse routes (103). IL-15 production is commonly associated with efficient control of blood brain barrier permeability in the extracellular nervous system fluid which is a hardly selective semipermeable border (101, 104, 105). Trans-activation of the IL-15 complex is a crucial factor in protecting against cerebral injuries in *Plasmodium*-infected mice through the induction of IL-10-producing NK cells (105). Therefore, one can assume that IL-15 may enable the host immune system to limit *T. rangeli* capacity for cell entry and eliminate the protozoan while it persists in the blood.

Conversely, *T. cruzi* has adopted countless strategies to manipulate the host immune response in order to survive (106, 107). During the acute phase of Chagas disease, in which the parasite is present in the bloodstream, an effective immune response is established to control parasite replication in the tissues (108) Production of interferon-γ, IL-17, and specific antibodies by NK, T, and B cells are crucial for parasite clearance. In mice, IL-17A deficiency impaired the activation of immune cells critical for the killing of *T. cruzi*, resulting in greater susceptibility of the mice to *T. cruzi* infection (109).

IL-17 impairment as a result of *T. cruzi* infection may represent a crucial mechanism of parasite immune evasion and consequent cellular infection. IL-17 is a key cytokine, produced mainly by T helper 17 cells (T_h_17) that promotes the recruitment of immune cells, including neutrophils and monocytes (110). IL-17A is required for the elimination of bacteria, fungi, and *T. cruzi*; it was found to be diminished in chagasic patients with established heart disease, especially in patients with congestive heart failure (111). We observed that the PSP DNAJA 2 leads to downregulation of IL-17 expression during pathogen invasion (Figure 3A). *T. cruzi* DNAJA 2 shows between 50–70% similarity to the human isoform. DNAJA 2 is related to the downregulation of RORα, one of the key signal transducers and activators of T_h_17 cells and consequent IL-17 production. Likewise, *in vivo* and *in vitro* studies showed that the absence of IL-17RA (IL-17 receptor)-signaling resulted in parasite-specific CD8+ T cell apoptosis, whereas recombinant IL-17A down-regulated the pro-apoptotic BAD protein and promoted the survival of activated CD8+ T cells (112). Furthermore, IL-17A production seems to serve an important immunomodulatory protective role during human chronic Chagas disease, which correlates to improved left ventricular function and myocardial damage protection (111). These effects may also be observed in benznidazole-treated individuals with increased IL-17 blood levels (113).

## Conclusion

PSPs from both *T. cruzi* and *T. ran*geli, which are pathogenic and non-pathogenic to humans, respectively, have been identified through the development of an *in silico* secretome. A hypothetical hybrid interactome of PSPs revealed that *T. rangeli* could enhance the production of IL-15, leading to NF-kB complex activation, and ultimately an immune response, which could explain the inability of *T. rangeli* to establish human infection. Conversely, *T. cruzi* could secrete proteins that trigger a decrease in IL-17. IL-17A is required for the elimination of bacteria, fungi, and *T. cruzi*, and it is diminished in chagasic patients with established heart disease. These results suggest that key PSPs could be used as novel therapeutic targets to minimize the health consequences of Chagas disease by subverting immune response-triggered by *T. cruzi* infection.

## Data Availability Statement

The datasets presented in this study can be found in online repositories. The names of the repository/repositories and accession number(s) can be found in the article/Supplementary Material.

## Author Contributions

DB conceived the study. RV, LL, LF, FA, and DB designed the experiments. RW, MB, IM, RV, CN, AM, IA-L, LL, and LF performed the experiments. RW, MB, IM, JS, LL, FA, and DB interpreted the results and analyzed the data. RV, LF, and FA contributed with analyses tools. RW, MB, IM, and DB wrote the manuscript. All authors read and approved the final manuscript.

## Conflict of Interest

The authors declare that the research was conducted in the absence of any commercial or financial relationships that could be construed as a potential conflict of interest.

## Supplementary Material

Supplementary Figure 1 Computational analysis workflow. SecretomeP 1.0, Psort II, Prop 1.0, SignalP 4.1, TMHMM 2.0, Gnuplot 4.0, and TargetP were kindly provided by Center Biological Sequence Analysis (CBS); Verify_proteins and Organize_table are in house perl scripts developed to parse software results and organize the results.

Supplementary Figure 2 *Trypanosoma cruzi* Sylvio X10/1-2012 and *T. rangeli* PSPs annotation. PSPs and their respective quantities found (n) in descending order. Proteins with only *n* = 1 were grouped.

Supplementary Table 1 Annotation of PSPs of both *T. cruzi* Sylvio and *T. rangeli*. Hypothetical proteins have been annotated manually. In blue, proteins selected to perform human interactome; in yellow protein used to phylogenetic tree. In green, Hsp70 proteins found in both species. TR04358 is a gene of *T. rangeli* genome that has not been deposited in TriTrypDB.

Supplementary Table 2 Manual classification of *T. cruzi* Sylvio PSPs according to functional categories.

Supplementary Table 3 Manual classification of *T. rangeli* PSPs according to functional categories.

Supplementary Table 4 Quantitative and qualitative analysis by OrthoMCL. Amount of grouped proteins found in each organism and in both species.

Supplementary Table 5 Identification of PSPs in other *T. cruzi* strains, compared to PSPs of *T. cruzi* Sylvio X10/1-2012. *T. cruzi* Sylvio (TcI) PSPs are used as queries to find orthologs in other strains such as Dm28 (TcI), Cl Brener Esmeraldo-like (TcVI), Cl Brener Non-Esmeraldo-like (TcVI), and TCC TcVI). A manual BLASTP was performed using TriTrypDB, and the presence of signal peptide (SP) and transmembrane domains (TM) has been evaluated by Interpro domain embedded in TriTrypDB. Proteins with *E-*value lower than 0 (negative value) were considered. When two or more copies of the same protein were found, the copy with the lowest *E-*value was considered. SP+ = presence of signal peptide; TM+ = presence of transmembrane helices; SP+ TM– means that it is considered a PSP (absence of transmembrane helices and presence of signal peptide); “–” symbol means that ortholog was not found.

Supplementary Table 6 Sequences producing significant alignments with Patatin sequence of *T. cruzi* Sylvio (TCSYLVIO_003749).

Supplementary Table 7 Aminoacid substitutions models.

## References

[1] Stevens JR Noyes HA Schofield CJ. Gibson W. The molecular evolution of trypanosomatidae. Adv Parasitol. (2001) 48:1–56. 10.1016/S0065-308X(01)48003-1 11013754

[2] Grisard EC Steindel M Guarneri AA Eger-Mangrich I Campbell DA Romanha AJ. Characterization of *Trypanosoma rangeli* strains isolated in central and south America: an overview. Mem Inst Oswaldo Cruz. (1999) 94:203–9. 10.1590/S0074-02761999000200015 10224529

[3] Espinosa-Álvarez O Ortiz PA Lima L Costa-Martins AG Serrano MG Herder S. *Trypanosoma rangeli* is phylogenetically closer to old world trypanosomes than to *Trypanosoma cruzi*. Int J Parasitol. (2018) 48:569–84. 10.1016/j.ijpara.2017.12.008 29544703

[4] Pérez-Molina JA Molina I Chagas disease. Lancet. (2018) 391:82–94. 10.1016/S0140-6736(17)31612-4 28673423

[5] Lima FM Oliveira P Mortara RA Silveira JF Bahia D. The challenge of chagas' disease: has the human pathogen, *Trypanosoma cruzi*, learned how to modulate signaling events to subvert host cells? N Biotechnol. (2010) 27:837–43. 10.1016/j.nbt.2010.02.003 20172059

[6] Nunes MCP Beaton A Acquatella H Bern C Bolger AF Echeverría LE. Chagas cardiomyopathy: an update of current clinical knowledge and management: a scientific statement from the american heart association. Circulation. (2018) 138:169–209. 10.1161/CIR.0000000000000599 30354432

[7] de Moraes MH Guarneri AA Girardi FP Rodrigues JB Eger I Tyler KM Different serological cross-reactivity of *Trypanosoma rangeli* forms in *Trypanosoma cruzi*-infected patient's sera. Parasit Vectors. (2008) 1:20 10.1186/1756-3305-1-20 18611261 PMC2475519

[8] Stoco PH Wagner G Talavera-Lopez C Gerber A Zaha A Thompson CE. Genome of the avirulent human-infective trypanosome *Trypanosoma rangeli*. PLoS Negl Trop Dis. (2014) 8:e3176. 10.1371/journal.pntd.0003176 25233456 PMC4169256

[9] Grisard EC Stoco PH Wagner G Sincero TC Rotava G Rodrigues JB. Transcriptomic analyses of the avirulent protozoan parasite *Trypanosoma rangeli*. Mol Biochem Parasitol. (2010) 174:18–25. 10.1016/j.molbiopara.2010.06.008 20600354

[10] Watanabe Costa R da Silveira JF Bahia D. Interactions between *Trypanosoma cruzi* secreted sroteins and host cell signaling pathways. Front Microbiol. (2016) 7:388. 10.3389/fmicb.2016.00388 27065960 PMC4814445

[11] Bayer-SantosEAguilar-BonavidesCRodriguesSPCorderoEMMarquesAFVarela-RamirezA. Proteomic analysis of *Trypanosoma cruzi* secretome: characterization of two populations of extracellular vesicles and soluble proteins. J Proteome Res. (2013) 12:883–97. 10.1021/pr300947g23214914

[12] Aslett M Aurrecoechea C Berriman M Brestelli J Brunk BP Carrington M. TriTrypDB: a functional genomic resource for the trypanosomatidae. Nucleic Acids Res. (2010) 38:D457–62. 10.1093/nar/gkp851 19843604 PMC2808979

[13] Nielsen H Engelbrecht J Brunak S Von Heijne G. A neural network method for identification of prokaryotic and eukaryotic signal peptides and prediction of their cleavage sites. Int J Neural Syst. (1997) 8:581–99. 10.1142/S0129065797000537 10065837

[14] Bendtsen JD Jensen LJ Blom N Von Heijne G Brunak S. Feature based prediction of non-classical and leaderless protein secretion. Protein Eng Des Sel. (2004) 17:349–56. 10.1093/protein/gzh037 15115854

[15] Krogh A Larsson B von Heijne G Sonnhammer EL. Predicting transmembrane protein topology with a hidden Markov model: Application to complete genomes. J Mol Biol. (2001) 305:567–80. 10.1006/jmbi.2000.4315 11152613

[16] Tusnady GE Simon I. Principles governing amino acid composition of integral membrane proteins: application to topology prediction. J Mol Biol. (1998) 283:489–506. 10.1006/jmbi.1998.2107 9769220

[17] Tusnady GE Simon I. The HMMTOP transmembrane topology prediction server. Bioinformatics. (2001) 17:849–50. 10.1093/bioinformatics/17.9.849 11590105

[18] Hofmann K Stoffel W TMbase - A database of membrane spanning proteins segments. Biol Chem Hoppe Seyler. (1993) 374:166.

[19] Cserzö M Eisenhaber F Eisenhaber B Simon I. TM or not TM: transmembrane protein prediction with low false positive rate using DAS-Tmfilter. Bioinformatics. (2004) 20:136–7. 10.1093/bioinformatics/btg394 14693825

[20] Götz S García-Gómez JM Terol J Williams TD Nagaraj SH Nueda MJ. High-throughput functional annotation and data mining with the Blast2GO suite. Nucleic Acids Res. (2008) 36:3420–35. 10.1093/nar/gkn176 18445632 PMC2425479

[21] NCBI Resource Coordinators Database resources of the National Center for Biotechnology Information. Nucleic Acids Res. (2018) 46:D8–13. 10.1093/nar/gkx1095 29140470 PMC5753372

[22] The UniProt Consortium. UniProt: a worldwide hub of protein knowledge. Nucleic Acids Res. (2019) 47:D506–15. 10.1093/nar/gky1049 30395287 PMC6323992

[23] Boeckmann B Bairoch A Apweiler R Blatter MC Estreicher A Gasteiger E. The Swiss-Prot protein knowledgebase and its supplement TrEMBL. Nucleic Acids Res. (2003) 31:365–70. 10.1093/nar/gkg095 12520024 PMC165542

[24] O'Leary NA Wright MW Brister JR Ciufo S Haddad D McVeigh R. Reference sequence (RefSeq) database at NCBI: current status, taxonomic expansion, functional annotation. Nucleic Acids Res. (2016) 44:D733–45. 10.1093/nar/gkv1189 26553804 PMC4702849

[25] Greenwell BM pdb: An R Package for Constructing Partial Dependence Plots. R J. (2017) 9:421–36. 10.32614/RJ-2017-016

[26] Jones P Binns D Chang HY Fraser M Li W McAnulla C. InterProScan 5: genome-scale protein function classification. Bioinformatics. (2014) 30:1236–40. 10.1093/bioinformatics/btu031 24451626 PMC3998142

[27] Chen F Mackey AJ Stoeckert CJ Jr Roos DS. OrthoMCL-DB: querying a comprehensive multi-species collection of ortholog groups. Nucleic Acids Res. (2006) 34:D363–8. 10.1093/nar/gkj123 16381887 PMC1347485

[28] Christian LJ Stoeckert CJ Jr Roos DS. OrthoMCL: identification of ortholog groups for eukaryotic genomes. Genome Res. (2003) 13:2178–89. 10.1101/gr.1224503 12952885 PMC403725

[29] Chen F Mackey AJ Vermunt JK Roos DS. Assessing performance of orthology detection strategies applied to eukaryotic genomes. PLoS ONE. (2007) 2:e383. 10.1371/journal.pone.0000383 17440619 PMC1849888

[30] El-Gebali S Mistry J Bateman A Eddy SR Luciani A Potter SC. The Pfam protein families database in 2019. Nucleic Acids Res. (2019) 47:D427–32. 10.1093/nar/gky995 30357350 PMC6324024

[31] Morgulis A Coulouris G Raytselis Y Madden TL Agarwala R Schäffer AA. Database indexing for production MegaBLAST searches. Bioinformatics. (2008) 15:1757–64. 10.1093/bioinformatics/btn322 18567917 PMC2696921

[32] Edgar RC. MUSCLE: multiple sequence alignment with high accuracy and high throughput. Nucleic Acids Res. (2004) 32:1792–7. 10.1093/nar/gkh340 15034147 PMC390337

[33] Galtier N Gouy M Gautier C. SEAVIEW and PHYLO_WIN: two graphic tools for sequence alignment and molecular phylogeny. Comput Appl Biosci. (1996) 12:543–8. 10.1093/bioinformatics/12.6.543 9021275

[34] Felsenstein J. Phylogenies from molecular sequences: Inference and reliability. Annu Rev Genet. (1988) 22:521–65. 10.1146/annurev.ge.22.120188.002513 3071258

[35] Ronquist F Teslenko M van der Mark P Ayres DL Darling A Hohna S. MrBayes 3.2: efficient Bayesian phylogenetic inference and model choice across a large model space. Syst Biol. (2012) 61:539–42. 10.1093/sysbio/sys029 22357727 PMC3329765

[36] Le SQ Gascuel O. An improved general amino acid replacement matrix. Mol Biol Evol. (2008) 25:1307–20. 10.1093/molbev/msn067 18367465

[37] Tamura K Stecher G Peterson D Filipski A Kumar S. MEGA6: molecular evolutionary genetics analysis version 6.0. Mol Biol Evol. (2013) 30:2725–9. 10.1093/molbev/mst197 24132122 PMC3840312

[38] Su MG Huang KY Lu CT Kao HJ Chang YH Lee TY. topPTM: a new module of dbPTM for identifying functional post-translational modifications in transmembrane proteins. Nucleic Acids Res. (2014) 42:D537–45. 10.1093/nar/gkt1221 24302577 PMC3965085

[39] Epting CL Coates BM Engman DM. Molecular mechanisms of host cell invasion by *Trypanosoma cruzi*. Exp Parasitol. (2010) 126:283–91. 10.1016/j.exppara.2010.06.023 20599990 PMC3443968

[40] El-Sayed NM Myler PJ Bartholomeu DC Nilsson D Aggarwal G Tran A. The genome sequence of *Trypanosoma cruzi*, etiologic agent of Chagas disease. Science. (2005) 309:409–15. 10.1126/science.1112631 16020725

[41] Bartholomeu DC Cerqueira GC Leão AC daRocha WD Pais FS Macedo C Genomic organization and expression profile of the mucin-associated surface protein (masp) family of the human pathogen *Trypanosoma cruzi*. Nucleic Acids Res. (2009) 7:3407–17. 10.1093/nar/gkp172 PMC2691823 19336417

[42] De Pablos LM Osuna A. Multigene families in *Trypanosoma cruzi* and their role in infectivity. Infect Immun. (2012) 80:2258–64. 10.1128/IAI.06225-11 22431647 PMC3416482

[43] Urban I Santurio LB Chidichimo A Yu H Chen X Mucci J. Molecular diversity of the *Trypanosoma cruzi* TcSMUG family of mucin genes and proteins. Biochem J. (2011) 438:303–13. 10.1042/BJ20110683 21651499

[44] Azuaje FJ Ramirez JL Da Silveira JF. *In silico*, biologically-inspired modelling of genomic variation generation in surface proteins of *Trypanosoma cruzi*. Kinetoplastid Biol Dis. (2007) 10:6–6. 10.1186/1475-9292-6-6 17623100 PMC1965468

[45] Cerqueira GC Bartholomeu DC DaRocha WD Hou L Freitas-Silva DM Machado CR. Sequence diversity and evolution of multigene families in *Trypanosoma cruzi*. Mol Biochem Parasitol. (2008) 157:65–72. 10.1016/j.molbiopara.2007.10.002 18023889

[46] Freitas LM dos Santos SL Rodrigues-Luiz GF Mendes TA Rodrigues TS Gazzinelli RT. Genomic analyses, gene expression and antigenic profile of the trans-sialidase superfamily of *Trypanosoma cruzi* reveal an undetected level of complexity. PLoS ONE. (2011) 6:e25914. 10.1371/journal.pone.0025914 22039427 PMC3198458

[47] Freire-de-Lima L Fonseca LM Oeltmann T Mendonça-Previato L Previato JO. The trans-sialidase, the major *Trypanosoma cruzi* virulence factor: Three decades of studies. Glycobiology. (2015) 25:1142–9. 10.1093/glycob/cwv057 26224786

[48] Wincker P Murto-Dovales AC Goldenberg S. Nucleotide sequence of a representative member of a *Trypanosoma cruzi* dispersed gene family. Mol Biochem Parasitol. (1992) 55:217–20. 10.1016/0166-6851(92)90142-7 1435871

[49] Atwood JA Minning T Ludolf F Nuccio A Weatherly DB Alvarez-Manilla G. Glycoproteomics of *Trypanosoma cruzi* Trypomastigotes Using Subcellular Fractionation, Lectin Affinity, and Stable Isotope Labeling. J Proteome Res. (2006) 5:3376–84. 10.1021/pr060364b 17137339

[50] Kawashita SY da Silva CV Mortara RA Burleigh BA Briones MR. Homology, paralogy and function of DGF-1, a highly dispersed *Trypanosoma cruzi* specific gene family and its implications for information entropy of its encoded proteins. Mol Biochem Parasitol. (2009) 165:19–31. 10.1016/j.molbiopara.2008.12.010 19393159

[51] González AM Azuaje FJ Ramírez JL da Silveira JF Dorronsoro JR. Machine learning techniques for the automated classification of adhesin-like proteins in the human protozoan parasite *Trypanosoma cruzi*. IEEE/ACM Trans Comput Biol Bioinform. (2009) 6:695–702. 10.1109/TCBB.2008.125 19875867

[52] Lander N Bernal C Diez N Añez N Docampo R Ramírez JL. Localization and developmental regulation of a dispersed gene family 1 protein in *Trypanosoma cruzi*. Infect Immun. (2010) 78:231–40. 10.1128/IAI.00780-09 19841080 PMC2798230

[53] El-Sayed NM Donelson JE. African trypanosomes have differentially expressed genes encoding homologues of the leishmania GP63 surface protease. J Biol Chem. (1997) 272:26742–8. 10.1074/jbc.272.42.26742 9334260

[54] Cuevas IC Cazzulo JJ Sánchez DO. gp63 homologues in *Trypanosoma cruzi*: surface antigens with metalloprotease activity and a possible role in host cell infection. Infect Immun. (2003) 71:5739–49. 10.1128/IAI.71.10.5739-5749.2003 14500495 PMC201075

[55] Alvarez VE Niemirowicz GT Cazzulo JJ. The peptidases of *Trypanosoma cruzi:* digestive enzymes, virulence factors, and mediators of autophagy and programmed cell death. Biochim Biophys Acta. (2012) 1824:195–206. 10.1016/j.bbapap.2011.05.011 21621652

[56] Llewellyn MS Messenger LA Luquetti AO Garcia L Torrico F Tavares SB. Deep sequencing of the *Trypanosoma cruzi* gp63 surface proteases reveals diversity and diversifying selection among chronic and congenital Chagas disease patients. PLoS Negl Trop Dis. (2015) 9:e0003458. 10.1371/journal.pntd.0003458 25849488 PMC4388557

[57] Baida RC Santos MR Carmo MS Yoshida N Ferreira D Ferreira AT. Molecular characterization of serine-, alanine-, and proline-rich proteins of *Trypanosoma cruzi* and their possible role in host cell infection. Infect. Immun. (2006) 74:1537–46. 10.1128/IAI.74.3.1537-1546.2006 16495524 PMC1418663

[58] Zanforlin T Bayer-Santos E Cortez C Almeida IC Yoshida N da Silveira JF Molecular characterization of *Trypanosoma cruzi* SAP proteins with host-cell lysosome exocytosis-inducing activity required for parasite invasion. PLoS ONE. (2013) 31:e83864 10.1371/journal.pone.0083864 PMC3877114 24391838

[59] dos Santos SL Freitas LM Lobo FP Rodrigues-Luiz GF Mendes TAO Oliveira ACS. The MASP family of *Trypanosoma cruzi*: changes in gene expression and antigenic profile during the acute phase of experimental infection. PLoS Negl Trop Dis. (2012) 6:e1779. 10.1371/journal.pntd.0001779 22905275 PMC3419193

[60] Serna C Lara JA Rodrigues SP Marques AF Almeida IC Maldonado RA. A synthetic peptide from *Trypanosoma cruzi* mucin-like associated surface protein as candidate for a vaccine against Chagas disease. Vaccine. (2014) 32:3525–32. 10.1016/j.vaccine.2014.04.026 24793944 PMC4058865

[61] De Pablos LM Díaz Lozano IM Jercic MI Quinzada M Giménez MJ Calabuig E. The C-terminal region of *Trypanosoma cruzi* MASPs is antigenic and secreted via exovesicles. Sci Rep. (2016) 6:27293. 10.1038/srep27293 27270330 PMC4897614

[62] Díaz Lozano IM De Pablos LM Longhi SA Zago MP Schijman AG Osuna A. Immune complexes in chronic Chagas disease patients are formed by exovesicles from *Trypanosoma cruzi* carrying the conserved MASP N-terminal region. Sci Rep. (2017) 7:44451. 10.1038/srep44451 28294160 PMC5353755

[63] Maeda FY Cortez C Yoshida N. Cell signaling during *Trypanosoma cruzi* invasion. Front Immunol. (2012) 3:361. 10.3389/fimmu.2012.00361 23230440 PMC3515895

[64] Atwood JA Weatherly DB Minning TA Bundy B Cavola C Opperdoes FR. The *Trypanosoma cruzi* proteome. Science. (2005) 309:473–6. 10.1126/science.1110289 16020736

[65] Buscaglia CA Campo VA Frasch AC Di Noia JM. *Trypanosoma cruzi* surface mucins: host-dependent coat diversity. Nat Rev Microbiol. (2006) 4:229–36. 10.1038/nrmicro1351 16489349

[66] Pereira-Chioccola VL Acosta-Serrano A Correia de Almeida I Ferguson MA Souto-Padron MM Travassos LR. Mucin-like molecules form a negatively charged coat that protects *Trypanosoma cruzi* trypomastigotes from killing by human anti-alpha-galactosyl antibodies. J Cell Sci. (2000) 113:1299–307. 10704380 10.1242/jcs.113.7.1299

[67] Schenkman S Ferguson MA Heise N de Almeida ML Mortara RA Yoshida N. Mucin-like glycoproteins linked to the membrane by glycosylphosphatidylinositol anchor are the major acceptors of sialic acid in a reaction catalyzed by trans-sialidase in metacyclic forms of *Trypanosoma cruzi*. Mol Biochem Parasitol. (1993) 59:293–303. 10.1016/0166-6851(93)90227-O 8341326

[68] Kulkarni MM Olson CL Engman DM McGwire BS. *Trypanosoma cruzi* gp63 proteins undergo stage-specific differential posttranslational modification and are important for host cell infection. Infect Immun. (2009) 77:2193–200. 10.1128/IAI.01542-08 19273559 PMC2681764

[69] Asea A Rehli M Kabingu E Boch JA Bare O Auron PE. Novel signal transduction pathway utilized by extracellular HSP70: role of toll-like receptor (TLR) 2 and TLR4. J Biol Chem. (2002) 277:15028–34. 10.1074/jbc.M200497200 11836257

[70] Cuellar A Santander SP Thomas M del C Guzmán F Gómez A. Monocyte-derived dendritic cells from chagasic patients vs healthy donors secrete differential levels of IL-10 and IL-12 when stimulated with a protein fragment of *Trypanosoma cruzi* heat-shock protein-70. Immunol Cell Biol. (2008) 86:255–60. 10.1038/sj.icb.7100146 18180802

[71] Flechas ID Cuellar A Cucunubá ZM Rosas Velasco FV Steindel M. Characterising the KMP-11 and HSP-70 recombinant antigens' humoral immune response profile in chagasic patients. BMC Infect Dis. (2009) 9:186. 10.1186/1471-2334-9-186 19939275 PMC2789076

[72] Grandgenett PM Coughlin BC Kirchhoff LV Donelson JE. Differential expression of gp63 genes in *Trypanosoma cruzi*. Mol Biochem Parasitol. (2000) 110:409–15. 10.1016/S0166-6851(00)00275-9 11071294

[73] Yamanaka CN Giordani RB Rezende CO Eger I Kessler RL Tonini ML. The *Trypanosoma rangeli* trypomastigote surfaceome reveals novel proteins and targets for specific diagnosis. J Proteome. (2013) 82:52–63. 10.1016/j.jprot.2013.02.011 23466310

[74] Ferreira ÉR Horjales E Bonfim-Melo A Cortez C da Silva CV De Groote M. Unique behavior of *Trypanosoma cruzi* mevalonate kinase: a conserved glycosomal enzyme involved in host cell invasion and signaling. Sci Rep. (2016) 6:24610. 10.1038/srep24610 27113535 PMC4845012

[75] Bahia D. A new trick for a conserved enzyme: mevalonate kinase, a glycosomal enzyme, can be secreted by *Trypanosoma cruzi* and modulate cell invasion and signaling. Is it another moonlighting enzyme? Front Cell Infect Microbiol. (2017) 7:426. 10.3389/fcimb.2017.00426 29034216 PMC5627032

[76] Oliveira MT Branquinho RT Alessio GD Mello CGC Nogueira-de-Paiva NC Carneiro CM. TcI, TcII and TcVI *Trypanosoma cruzi* samples from Chagas disease patients with distinct clinical forms and critical analysis of *in vitro* and *in vivo* behavior, response to treatment and infection evolution in murine model. Acta Trop. (2017) 167:108–20. 10.1016/j.actatropica.2016.11.033 27908747

[77] Brossas JY Gulin JEN Bisio MMC Chapelle M Marinach-Patrice C Bordessoules M. Secretome analysis of *Trypanosoma cruzi* by proteomics studies. PLoS ONE. (2017) 12:e0185504. 10.1371/journal.pone.0185504 28972996 PMC5626432

[78] Belaunzarán ML Wainszelbaum MJ Lammel EM Gimenez G Aloise MM Florin-Christensen J. Phospholipase A1 from *Trypanosoma cruzi* infective stages generates lipid messengers that activate host cell protein kinase C. Parasitology. (2007) 134:491–502. 10.1017/S0031182006001740 17121684

[79] Belaunzarán ML Wilkowsky SE Lammel EM Giménez G Bott E Barbieri MA. Phospholipase A1: a novel virulence factor in *Trypanosoma cruzi*. Mol Biochem Parasitol. (2013) 187:77–86. 10.1016/j.molbiopara.2012.12.004 23275096

[80] Marchler-Bauer A Derbyshire MK Gonzales NR Lu S Chitsaz F Geer LY. CDD: NCBI's conserved domain database. Nucleic Acids Res. (2015) 43:D222–6. 10.1093/nar/gku1221 25414356 PMC4383992

[81] Dekker SL Kampinga HH Bergink S. DNAJs: more than substrate delivery to HSPA. Front Mol Biosci. (2015) 2:35. 10.3389/fmolb.2015.00035 26176011 PMC4485348

[82] Folgueira C Requena JM. A postgenomic view of the heat shock proteins in kinetoplastids. FEMS Microbiol Rev. (2007) 31:359–77. 10.1111/j.1574-6976.2007.00069.x 17459115

[83] Shonhai A Maier AG Przyborski JM Blatch GL. Intracellular protozoan parasites of humans: the role of molecular chaperones in development and pathogenesis. Protein Pept Lett. (2011) 18:143–57. 10.2174/092986611794475002 20955165

[84] Urményi TP Silva R Rondinelli E. The heat shock proteins of *Trypanosoma cruzi*. Subcell Biochem. (2014) 74:119–35. 10.1007/978-94-007-7305-9_5 24264243

[85] Real F Vidal RO Carazzolle MF Mondego JM Costa GG Herai RH. The genome sequence of *Leishmania (Leishmania) amazonensis*: functional annotation and extended analysis of gene models. DNA Res. (2013) 20:567–81. 10.1093/dnares/dst031 23857904 PMC3859324

[86] Mignery GA Pikaard CS Park WD. Molecular characterization of the patatin multigene family of potato. Gene. (1988) 62:27–44. 10.1016/0378-1119(88)90577-X 3371664

[87] Oliveira P Lima FM Cruz MC Ferreira RC Sanchez-Flores A Cordero EM. *Trypanosoma cruzi*: Genome characterization of phosphatidylinositol kinase gene family (PIK and PIK-related) and identification of a novel PIK gene, Infect. Genet Evol. (2014) 25:157–65. 10.1016/j.meegid.2014.03.022 24727645

[88] Caballero ZC Costa-Martins AG Ferreira RC P Alves JM Serrano MG Camargo EP. Phylogenetic and syntenic data support a single horizontal transference to a Trypanosoma ancestor of a prokaryotic proline racemase implicated in parasite evasion from host defences. Parasit Vectors. (2015) 8:222. 10.1186/s13071-015-0829-y 25890302 PMC4417235

[89] Fitzpatrick DA. Horizontal gene transfer in fungi. FEMS Microbiol Lett. (2012) 329:1–8. 10.1111/j.1574-6968.2011.02465.x 22112233

[90] Kidwell MG. Lateral transfer in natural populations of eukaryotes. Annu Rev Genet. (1993) 27:235–56. 10.1146/annurev.ge.27.120193.001315 8122903

[91] Ravenhall M Škunca N Lassalle F Dessimoz C. Inferring horizontal gene transfer. PLoS Comput Biol. (2015) 11:e1004095. 10.1371/journal.pcbi.1004095 26020646 PMC4462595

[92] de Carvalho MO Loreto EL. Methods for detection of horizontal transfer of transposable elements in complete genomes. Genet Mol Biol. (2012) 35:1078–84. 10.1590/S1415-47572012000600024 23411916 PMC3571429

[93] Añez N Velandia J Rodríguez AM. *Trypanosoma rangeli* Tejera, 1920. VIII. Response to reinfections in 2 mammals. Mem Inst Oswaldo Cruz. (1985) 80:149–53. 10.1590/S0074-02761985000200005 3836326

[94] Steindel M Dias Neto E de Menezes CL Romanha AJ Simpson AJ. Random amplified polymorphic DNA analysis of *Trypanosoma cruzi* strains. Mol Biochem Parasitol. (1993) 60:71–9. 10.1016/0166-6851(93)90030-2 8366896

[95] Osorio Y Travi BL Palma GI Saravia NG. Infectivity of *Trypanosoma rangeli* in a promonocytic mammalian cell line. J Parasitol. (1995) 81:687–93. 10.2307/3283955 7472856

[96] Eger-Mangrich I de Oliveira MA Grisard EC De Souza W Steindel M. Interaction of *Trypanosoma rangeli* Tejera, 1920 with different cell lines *in vitro*. Parasitol Res. (2001) 87:505–9. 10.1007/s004360000356 11411954

[97] Zuñiga C Palau T Penin P Gamallo C De Diego JA. Protective effect of *Trypanosoma rangeli* against infections with a highly virulent strain of *Trypanosoma cruzi*. Trop Med Int Health. (1997) 2:482–7. 10.1046/j.1365-3156.1997.d01-297.x 9217704

[98] de Sousa MA Dos Santos Pereira SM Dos Santos Faissal BN. Variable sensitivity to complement-mediated lysis among *Trypanosoma rangeli* reference strains. Parasitol Res. (2012) 110:599–608. 10.1007/s00436-011-2528-8 21748348

[99] Yatim KM Lakkis FG. A brief journey through the immune system. Clin J Am Soc Nephrol. (2015) 10:1274–81. 10.2215/CJN.10031014 25845377 PMC4491295

[100] Eberl G. Immunity by equilibrium. Nat Rev Immunol. (2016) 16:524–32. 10.1038/nri.2016.75 27396446

[101] Stone KP Kastin AJ Pan W. NFκB is an unexpected major mediator of interleukin-15 signaling in cerebral endothelia. Cell Physiol Biochem. (2011) 28:115–24. 10.1159/000331720 21865854 PMC3709181

[102] Ring AM Lin JX Feng D Mitra S Rickert M Bowman GR. Mechanistic and structural insight into the functional dichotomy between IL-2 and IL-15. Nat Immunol. (2012)13:1187–95. 10.1038/ni.2449 23104097 PMC3501574

[103] Jabri B Abadie V. IL-15 functions as a danger signal to regulate tissue-resident T cells and tissue destruction. Nat Rev Immunol. (2015) 15:771–83. 10.1038/nri3919 26567920 PMC5079184

[104] Stone KP Kastin AJ Hsuchou H Yu C Pan W. Rapid endocytosis of interleukin-15 by cerebral endothelia. J Neurochem. (2012) 116:544–53. 10.1111/j.1471-4159.2010.07142.x 21155807 PMC3076997

[105] Burrack KS Huggins MA Taras E Dougherty P Henzler CM Yang R. Interleukin-15 complex treatment protects mice from cerebral malaria by inducing interleukin-10-producing natural killer cells. Immunity. (2018) 48:760–72. 10.1016/j.immuni.2018.03.012 29625893 PMC5906161

[106] Soares-Silva M Diniz FF Gomes GN Bahia D. The mitogen-activated protein kinase (MAPK) pathway: Role in immune evasion by trypanosomatids. Front Microbiol. (2016) 7:183. 10.3389/fmicb.2016.00183 26941717 PMC4764696

[107] Cardoso MS Reis-Cunha JL Bartholomeu DC. Evasion of the Immune response by *Trypanosoma cruzi* during acute infection. Front Immunol. (2015) 6:659. 10.3389/fimmu.2015.00659 26834737 PMC4716143

[108] Cardillo F de Pinho RT Antas PR Mengel J. Immunity and immune modulation in *Trypanosoma cruzi* infection. Pathog Dis. (2015) 73:ftv082. 10.1093/femspd/ftv082 26438729 PMC4626602

[109] Miyazaki Y Hamano S Wang S Shimanoe Y Iwakura Yoshida YH. IL-17 is necessary for host protection against acute-phase *Trypanosoma cruzi* infection. J Immunol. (2010) 185:1150–7. 10.4049/jimmunol.0900047 20562260

[110] Weaver CT Elson CO Fouser LA Kolls JK. The Th17 pathway and inflammatory diseases of the intestines, lungs, and skin. Annu Rev Pathol Mech Dis. (2013) 8:477–512. 10.1146/annurev-pathol-011110-130318 23157335 PMC3965671

[111] Sousa GR Gomes JAS Damásio MPS Nunes MCP Costa HS Medeiros NI. The role of interleukin 17-mediated immune response in Chagas disease: high level is correlated with better left ventricular function. PLoS ONE. (2017) 12:e0172833. 10.1371/journal.pone.0172833 28278264 PMC5344340

[112] Tosello JB Araujo CLF Fiocca FV Vernengo FF Rodriguez C Ramello C. IL-17RA-signaling modulates CD8+ T cell survival and exhaustion during *Trypanosoma cruzi* infection. Front Immunol. (2018) 9:2347. 10.3389/fimmu.2018.02347 30364284 PMC6193063

[113] Camara EJN Mendonca VRR Souza LCL Carvalho JS Lessa RA Gatto R. Elevated IL-17 levels and echocardiographic signs of preserved myocardial function in benznidazole-treated individuals with chronic Chagas' disease. Int J Infect Dis. (2019) 79:123–30. 10.1016/j.ijid.2018.11.369 30528394

