# Supplementary figures and images for: Comparative Analysis of the Secretome and Interactome of Trypanosoma cruzi and Trypanosoma rangeli Reveals Species Specific Immune Response Modulating Proteins
Source: Front Immunol. 2020 Aug 27;11:1774. doi: 10.3389/fimmu.2020.01774 (PMC7481403; doi:10.3389/fimmu.2020.01774)

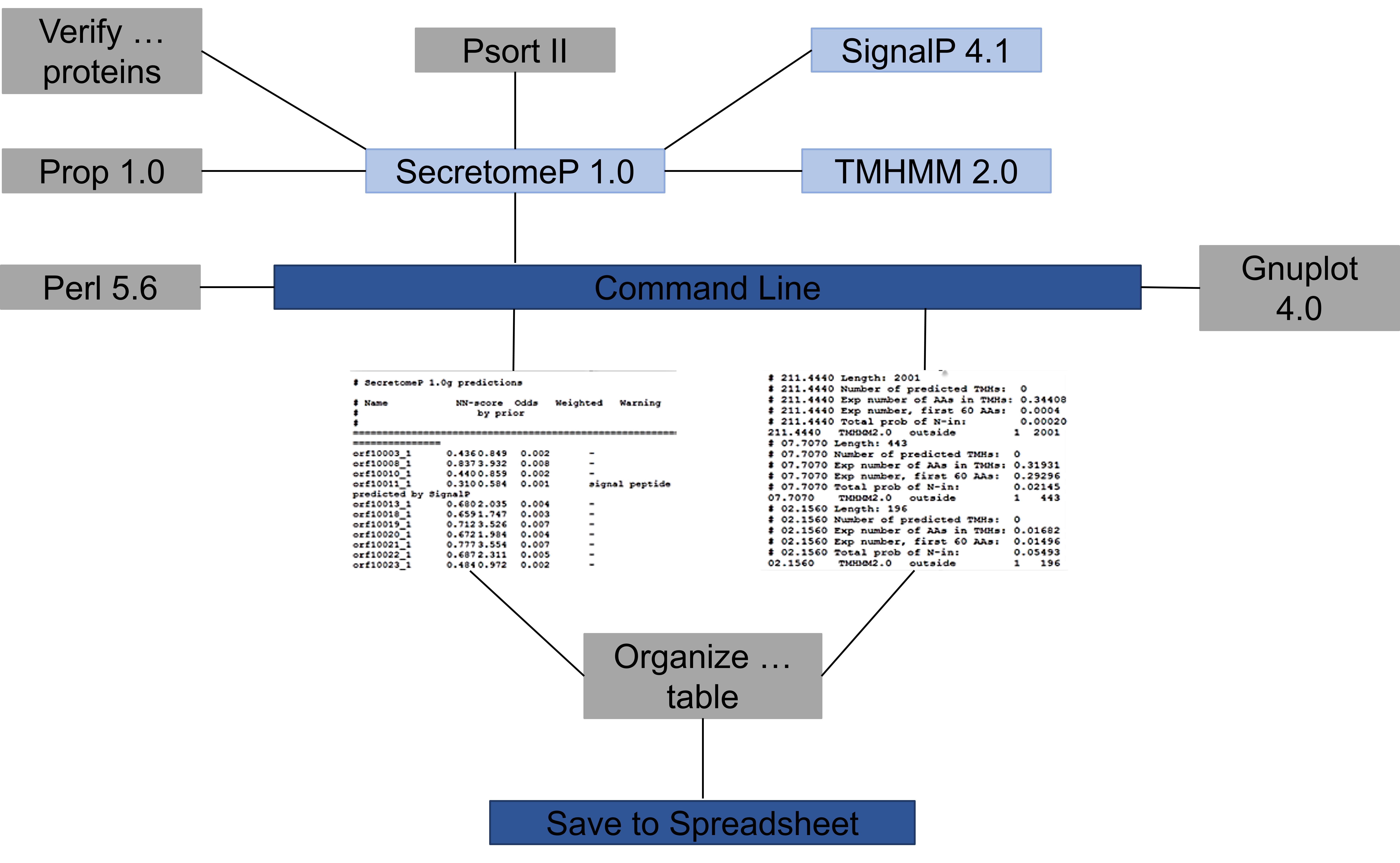

Supplement: Supplementary Figure 1 — Computational analysis workflow. SecretomeP 1.0, Psort II, Prop 1.0, SignalP 4.1, TMHMM 2.0, Gnuplot 4.0, and TargetP were kindly provided by Center Biological Sequence Analysis (CBS); Verify_proteins and Organize_table are in house perl scripts developed to parse software results and organize the results. [file Image_1.JPEG]

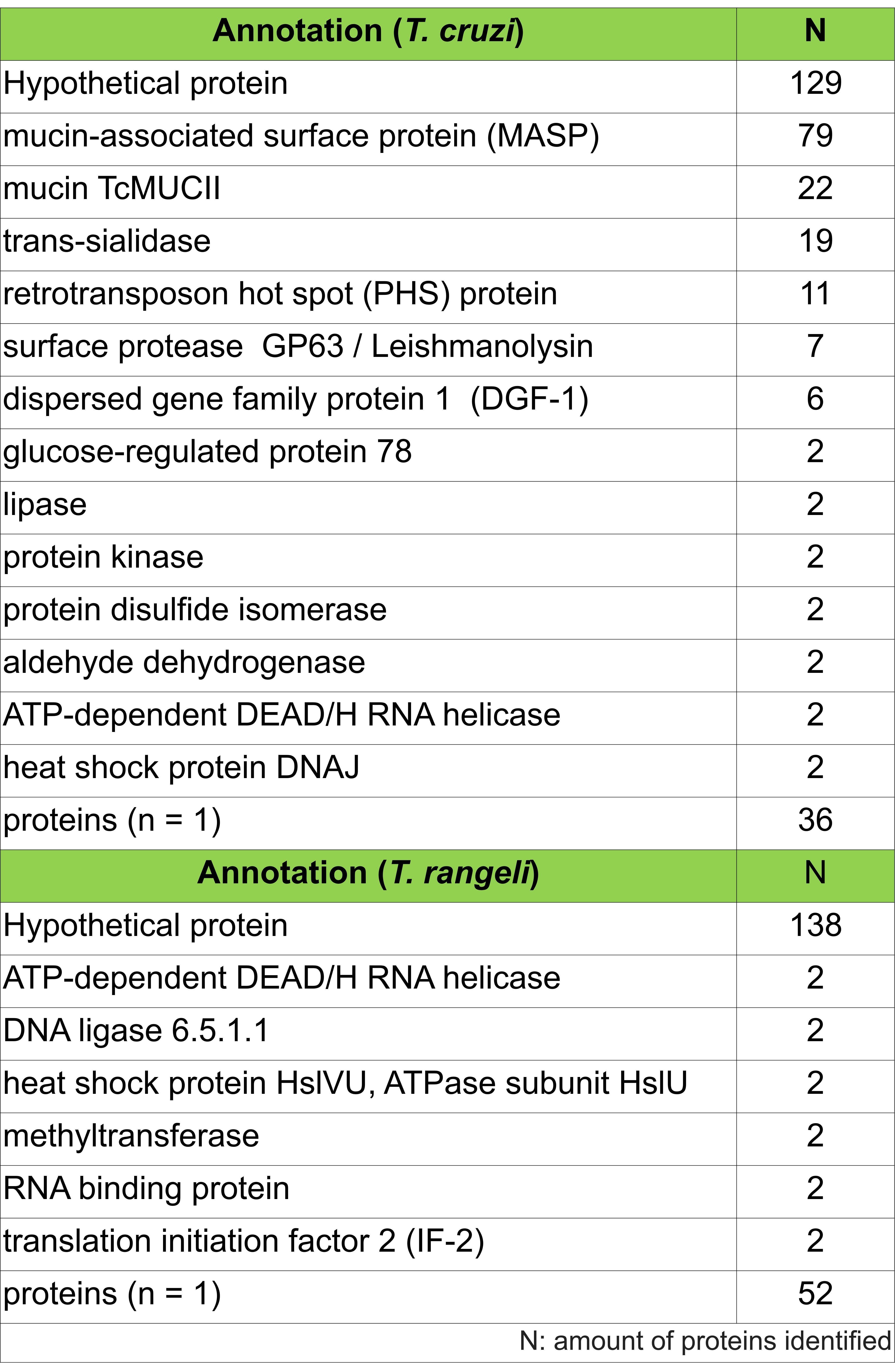

Supplement: Supplementary Figure 2 — Trypanosoma cruzi Sylvio X10/1-2012 and T. rangeli PSPs annotation. PSPs and their respective quantities found (n) in descending order. Proteins with only n = 1 were grouped. [file Image_2.JPEG]
