# Supplementary material for: Comparative Analysis of the Secretome and Interactome of Trypanosoma cruzi and Trypanosoma rangeli Reveals Species Specific Immune Response Modulating Proteins
Source: Front Immunol. 2020 Aug 27;11:1774. doi: 10.3389/fimmu.2020.01774 (PMC7481403; doi:10.3389/fimmu.2020.01774)
Supplement: Supplementary Table 4 — Quantitative and qualitative analysis by OrthoMCL. Amount of grouped proteins found in each organism and in both species. [file Table_4.DOCX]

| ***Trypanosoma cruzi* Sylvio** | | **Both species** | | ***Trypanosoma rangeli*** | |
| --- | --- | --- | --- | --- | --- |
| **ID** | **Annotation** | **ID** | **Annotation** | **ID** | **Annotation** |
| **Family: 1000** | | **Family: 1007** | | **Family: 1024** | |
| TCSYLVIO_009290 | trans-sialidase, putative | TCSYLVIO_003570 | surface protease GP63, putative | TRSC58_06631 | HP: mucin-associated surface protein (MASP) domain |
| TCSYLVIO_000676 | trans-sialidase, putative | TCSYLVIO_003765 | surface protease GP63, putative | TRSC58_07196 | HP: mucin-associated surface protein (MASP) domain |
| TCSYLVIO_002129 | trans-sialidase, putative | TCSYLVIO_003949 | surface protease GP63, putative | TRSC58_07137 | HP: mucin-associated surface protein (MASP) domain |
| TCSYLVIO_008550 | trans-sialidase, putative | TCSYLVIO_007929 | surface protease GP63, putative | **Family: 1037** | |
| TCSYLVIO_001127 | trans-sialidase, putative | TCSYLVIO_008659 | surface protease GP63, putative | TRSC58_01986 | *heat shock*protein HslVU, ATPase subunit HslU |
| TCSYLVIO_008305 | trans-sialidase, putative | TRSC58_06928 | Leishmanolysin | TRSC58_04483 | *heat shock*protein HslVU, ATPase subunit HslU |
| TCSYLVIO_008205 | trans-sialidase, putative | **Family: 1022** | | **Family: 1038** | |
| TCSYLVIO_000115 | trans-sialidase, putative | TCSYLVIO_007719 | glucose-regulated protein 78, putative | TRSC58_02069 | HP: phosphoenolpyruvate carboxykinase domain |
| TCSYLVIO_002490 | trans-sialidase, putative | TCSYLVIO_007845 | glucose-regulated protein 78, putative | TRSC58_02506 | HP: phosphoenolpyruvate carboxykinase domain |
| TCSYLVIO_000373 | trans-sialidase, putative | TRSC58_03733 | glucose-regulated protein 78, putative | **Family: 1039** | |
| TCSYLVIO_000115 | trans-sialidase, putative | **Family: 1054** | | TRSC58_03940 | HP: WD40 domain |
| TCSYLVIO_002490 | trans-sialidase, putative | TCSYLVIO_001780 | HP: DNA-binding transcriptional activator domain | TRSC58_04691 | HP: WD40 domain |
| TCSYLVIO_00373 | trans-sialidase, putative | TRSC58_03536 | hypothetical protein | **Family: 1040** | |
| TCSYLVIO_002150 | trans-sialidase, putative | **Family: 1055** | | TRSC58_05352 | 4-nitrophenyl phosphatase, putative |
| **Family: 1001** | | TCSYLVIO_001885 | serine/threonine protein phosphatase, putative | TRSC58_06433 | 4-nitrophenyl phosphatase, putative |
| TCSYLVIO_002201 | trans-sialidase, putative | TRSC58_01238 | serine/threonine protein phosphate, putative | **Family: 1053** | |
| TCSYLVIO_005139 | trans-sialidase, putative | **Family: 1056** | | TRSC58_04787 | hypothetical protein |
| TCSYLVIO_009000 | trans-sialidase, putative | TCSYLVIO_002105 | methyltransferase, putative | TRSC58_06966 | hypothetical protein |
| TCSYLVIO_008432 | trans-sialidase, putative | TRSC58_05399 | methyltransferase, putative |  |  |
| TCSYLVIO_007928 | trans-sialidase, putative | **Family: 1057** | |  |  |
| **Family: 1002** | | TCSYLVIO_002274 | DNA ligase, putative |  |  |
| TCSYLVIO_008171 | mucin TcMUCII, putative | TRSC58_04338 | DNA ligase, putative |  |  |
| TCSYLVIO_008962 | mucin TcMUCII, putative | **Family: 1058** | |  |  |
| TCSYLVIO_000263 | mucin TcMUCII, putative | TCSYLVIO_003660 | hypothetical protein: FAD-dependent. oxidoreductase domain |  |  |
| TCSYLVIO_008006 | mucin TcMUCII, putative | TRSC58_01549 | hypothetical protein: NAD(P)-binding domain |  |  |
| TCSYLVIO_006608 | mucin TcMUCII, putative | **Family: 1059** | |  |  |
| TCSYLVIO_005131 | mucin TcMUCII, putative | TCSYLVIO_003870 | hypothetical protein |  |  |
| TCSYLVIO_007946 | mucin TcMUCII, putative | TRSC58_00205 | hypothetical protein |  |  |
| TCSYLVIO_009659 | mucin TcMUCII, putative | **Family: 1060** | |  |  |
| TCSYLVIO_008321 | mucin TcMUCII, putative | TCSYLVIO_004057 | cytochrome-b5 reductase, putative |  |  |
| **Family: 1003** | | TRSC58_00139 | cytochrome-b5 reductase, putative |  |  |
| TCSYLVIO_010252 | retrotransposon hot spot protein, putative | **Family: 1061** | |  |  |
| TCSYLVIO_010804 | retrotransposon hot spot protein, putative | TCSYLVIO_004076 | hypothetical protein |  |  |
| TCSYLVIO_007954 | retrotransposon hot spot protein, putative | TRSC58_00855 | hypothetical protein |  |  |
| TCSYLVIO_002398 | retrotransposon hot spot protein, putative | **Family: 1062** | |  |  |
| TCSYLVIO_009618 | retrotransposon hot spot protein, putative | TCSYLVIO_004637 | legume-like lectin, putative |  |  |
| TCSYLVIO_009417 | retrotransposon hot spot protein, putative | TRSC58_00429 | legume-like lectin, putative |  |  |
| TCSYLVIO_002078 | retrotransposon hot spot protein, putative | **Family: 1063** | |  |  |
| TCSYLVIO_003055 | retrotransposon hot spot protein, putative | TCSYLVIO_000503 | hypothetical protein |  |  |
| TCSYLVIO_010841 | retrotransposon hot spot protein, putative | TRSC58_00436 | hypothetical protein: LD-Carboxypeptidase domain |  |  |
| **Family: 1004** | | **Family: 1064** | |  |  |
| TCSYLVIO_010388 | mucin-associated surface protein (MASP), putative | TCSYLVIO_005460 | NADH-cytochrome b5 reductase, putative |  |  |
| TCSYLVIO_008768 | mucin-associated surface protein (MASP), putative | TRSC58_03053 | NADH-cytochrome b5 reductase, putative |  |  |
| TCSYLVIO_008637 | mucin-associated surface protein (MASP), putative | **Family: 1065** | |  |  |
| TCSYLVIO_008339 | mucin-associated surface protein (MASP), putative | TCSYLVIO_005614 | RNA editing complex protein MP46, putative |  |  |
| TCSYLVIO_005257 | mucin-associated surface protein (MASP), putative | TRSC58_00967 | RNA editing complex protein MP46, putative |  |  |
| TCSYLVIO_007698 | mucin-associated surface protein (MASP), putative | **Family: 1066** | |  |  |
| TCSYLVIO_008109 | mucin-associated surface protein (MASP), putative | TCSYLVIO_005689 | hypothetical protein - *heat shock*70 kDa protein domain |  |  |
| TCSYLVIO_008972 | mucin-associated surface protein (MASP), putative | TRSC58_05509 | hypothetical protein - *heat shock*70 kDa protein domain |  |  |
| **Family: 1005** | | **Family: 1067** | |  |  |
| TCSYLVIO_009527 | mucin-associated surface protein (MASP), putative | TCSYLVIO_006554 | ATP-dependent DEAD/H RNA helicase, putative |  |  |
| TCSYLVIO_005227 | mucin-associated surface protein (MASP), putative | TRSC58_00643 | ATP-dependent DEAD/H RNA helicase, putative |  |  |
| TCSYLVIO_009006 | mucin-associated surface protein (MASP), putative | **Family: 1068** | |  |  |
| TCSYLVIO_008322 | mucin-associated surface protein (MASP), putative | TCSYLVIO_006828 | dihydrolipoamide acetyltransferase, putative |  |  |
| TCSYLVIO_005205 | mucin-associated surface protein (MASP), putative | TRSC58_00830 | dihydrolipoamide acetyltransferase, putative |  |  |
| TCSYLVIO_008822 | mucin-associated surface protein (MASP), putative | **Family: 1069** | |  |  |
| TCSYLVIO_005174 | mucin-associated surface protein (MASP), putative | TCSYLVIO_007253 | hypothetical protein |  |  |
| **Family: 1006** | | TRSC58_05094 | hypothetical protein |  |  |
| TCSYLVIO_007858 | mucin-associated surface protein (MASP), putative | **Family: 1070** | |  |  |
| TCSYLVIO_009535 | mucin-associated surface protein (MASP), putative | TCSYLVIO_007361 | hypothetical protein |  |  |
| TCSYLVIO_008450 | mucin-associated surface protein (MASP), putative | TRSC58_04309 | hypothetical protein |  |  |
| **Family: 1009** | | **Family: 1071** | |  |  |
| TCSYLVIO_008509 | mucin-associated surface protein (MASP), putative | TCSYLVIO_007462 | acid phosphatase, putative |  |  |
| **Family: 1010** | | TRSC58_05459 | acid phosphatase, putative |  |  |
| TCSYLVIO_010754 | mucin-associated surface protein (MASP), putative | **Family: 1073** | |  |  |
| TCSYLVIO_006589 | mucin-associated surface protein (MASP), putative | TCSYLVIO_000939 | ATP-dependent RNA helicase, putative |  |  |
| TCSYLVIO_006646 | mucin-associated surface protein (MASP), putative | TRSC58_00169 | ATP-dependent RNA helicase, putative |  |  |
| TCSYLVIO_006314 | mucin-associated surface protein (MASP), putative | **Family: 1074** | |  |  |
| TCSYLVIO_006583 | mucin-associated surface protein (MASP), putative | TCSYLVIO_00957 | cyclophilin, putative |  |  |
| **Family: 1011** | | TRSC58_02926 | cyclophilin, putative |  |  |
| TCSYLVIO_002216 | mucin-associated surface protein (MASP), putative |  |  |  |  |
| TCSYLVIO_006689 | mucin-associated surface protein (MASP), putative |  |  |  |  |
| TCSYLVIO_006684 | mucin-associated surface protein (MASP), putative |  |  |  |  |
| TCSYLVIO_006691 | mucin-associated surface protein (MASP), putative |  |  |  |  |
| TCSYLVIO_008118 | mucin-associated surface protein (MASP), putative |  |  |  |  |
| **Family: 1012** | |  |  |  |  |
| TCSYLVIO_005132 | mucin-associated surface protein (MASP), putative |  |  |  |  |
| TCSYLVIO_007735 | mucin-associated surface protein (MASP), putative |  |  |  |  |
| TCSYLVIO_009178 | mucin-associated surface protein (MASP), putative |  |  |  |  |
| TCSYLVIO_002032 | mucin-associated surface protein (MASP), putative |  |  |  |  |
| TCSYLVIO_000821 | mucin-associated surface protein (MASP), putative |  |  |  |  |
| **Family: 1013** | |  |  |  |  |
| TCSYLVIO_009562 | hypothetical protein |  |  |  |  |
| TCSYLVIO_010865 | 90 kDa surface protein, putative |  |  |  |  |
| **Family: 1014** | |  |  |  |  |
| TCSYLVIO_006694 | mucin-associated surface protein (MASP), putative |  |  |  |  |
| TCSYLVIO_006720 | mucin-associated surface protein (MASP), putative |  |  |  |  |
| **Family: 1015** | |  |  |  |  |
| TCSYLVIO_011009 | hypothetical protein |  |  |  |  |
| TCSYLVIO_006734 | hypothetical protein |  |  |  |  |
| TCSYLVIO_008382 | hypothetical protein |  |  |  |  |
| TCSYLVIO_006688 | hypothetical protein |  |  |  |  |
| **Family: 1016** | |  |  |  |  |
| TCSYLVIO_009252 | surface protease GP63, putative |  |  |  |  |
| **Family: 1018** | |  |  |  |  |
| TCSYLVIO_010251 | mucin-associated surface protein (MASP), putative |  |  |  |  |
| **Family: 1019** | |  |  |  |  |
| TCSYLVIO_005269 | mucin-associated surface protein (MASP), putative |  |  |  |  |
| **Family: 1020** | |  |  |  |  |
| TCSYLVIO_006643 | mucin-associated surface protein (MASP), putative |  |  |  |  |
| TCSYLVIO_007782 | mucin-associated surface protein (MASP), putative |  |  |  |  |
| TCSYLVIO_007925 | mucin-associated surface protein (MASP), putative |  |  |  |  |
| **Family: 1021** | |  |  |  |  |
| TCSYLVIO_006755 | mucin-associated surface protein (MASP), putative |  |  |  |  |
| TCSYLVIO_008438 | mucin-associated surface protein (MASP), putative |  |  |  |  |
| TCSYLVIO_000040 | mucin-associated surface protein (MASP), putative |  |  |  |  |
| **Family: 1023** | |  |  |  |  |
| TCSYLVIO_008045 | mucin-associated surface protein (MASP), putative |  |  |  |  |
| TCSYLVIO_009670 | mucin-associated surface protein (MASP), putative |  |  |  |  |
| **Family: 1025** | |  |  |  |  |
| TCSYLVIO_008155 | mucin-associated surface protein (MASP), putative |  |  |  |  |
| **Family: 1026** | |  |  |  |  |
| TCSYLVIO_010273 | dispersed gene family protein 1 (DGF-1), putative |  |  |  |  |
| TCSYLVIO_010417 | dispersed gene family protein 1 (DGF-1), putative |  |  |  |  |
| **Family: 1028** | |  |  |  |  |
| TCSYLVIO_011084 | lipase, putative |  |  |  |  |
| TCSYLVIO_009850 | lipase, putative |  |  |  |  |
| **Family: 1029** | |  |  |  |  |
| TCSYLVIO_002346 | protein disulfide isomerase, putative |  |  |  |  |
| TCSYLVIO_004580 | protein disulfide isomerase, putative |  |  |  |  |
| **Family: 1030** | |  |  |  |  |
| TCSYLVIO_003320 | aldehyde dehydrogenase, putative |  |  |  |  |
| TCSYLVIO_000041 | aldehyde dehydrogenase, putative |  |  |  |  |
| **Family: 1031** | |  |  |  |  |
| TCSYLVIO_003908 | hypothetical protein |  |  |  |  |
| TCSYLVIO_008963 | hypothetical protein |  |  |  |  |
| **Family: 1032** | |  |  |  |  |
| TCSYLVIO_008640 | mucin-associated surface protein (MASP), putative |  |  |  |  |
| **Family: 1033** | |  |  |  |  |
| TCSYLVIO_006610 | mucin TcMUCII, putative |  |  |  |  |
| TCSYLVIO_006724 | mucin TcMUCII, putative |  |  |  |  |
| **Family: 1034** | |  |  |  |  |
| TCSYLVIO_007837 | mucin-associated surface protein (MASP), putative |  |  |  |  |
| TCSYLVIO_008663 | mucin-associated surface protein (MASP), putative |  |  |  |  |
| **Family: 1035** | |  |  |  |  |
| TCSYLVIO_008310 | mucin-associated surface protein (MASP), putative |  |  |  |  |
| TCSYLVIO_008517 | mucin-associated surface protein (MASP), putative |  |  |  |  |
| **Family: 1042** | |  |  |  |  |
| TCSYLVIO_010755 | mucin TcMUCII, putative |  |  |  |  |
| TCSYLVIO_008736 | mucin-associated surface protein (MASP), putative |  |  |  |  |
| **Family: 1043** | |  |  |  |  |
| TCSYLVIO_011075 | mucin-associated surface protein (MASP), putative |  |  |  |  |
| TCSYLVIO_009491 | trans-sialidase, putative |  |  |  |  |
| **Family: 1044** | |  |  |  |  |
| TCSYLVIO_002224 | mucin-associated surface protein (MASP), putative |  |  |  |  |
| TCSYLVIO_008024 | mucin-associated surface protein (MASP), putative |  |  |  |  |
| **Family: 1045** | |  |  |  |  |
| TCSYLVIO_002984 | UDP-Gal/UDP-GlcNAc-depend. glycosyltransferase, putative |  |  |  |  |
| **Family: 1046** | |  |  |  |  |
| TCSYLVIO_000403 | surface protein TolT, putative |  |  |  |  |
| **Family: 1047** | |  |  |  |  |
| TCSYLVIO_005847 | chaperone DNAJ protein, putative |  |  |  |  |
| TCSYLVIO_005909 | heat shock protein DNAJ, putative |  |  |  |  |
| **Family: 1048** | |  |  |  |  |
| TCSYLVIO_000773 | dispersed gene family protein 1 (DGF-1), putative |  |  |  |  |
| TCSYLVIO_008521 | dispersed gene family protein 1 (DGF-1), putative |  |  |  |  |
| **Family: 1049** | |  |  |  |  |
| TCSYLVIO_008073 | mucin-associated surface protein (MASP), putative |  |  |  |  |
| **Family: 1052** | |  |  |  |  |
| TCSYLVIO_000923 | mucin TcMUCII, putative |  |  |  |  |
| TCSYLVIO_000936 | mucin TcMUCII, putative |  |  |  |  |
| **TOTAL** | **123** |  | **49** |  | **13** |
